# Development and *in vivo* evaluation of ^18^F-labeled PET tracers covalently targeting KRAS-G12C for noninvasive cancer diagnosis and therapy monitoring

**DOI:** 10.7150/thno.123741

**Published:** 2026-05-01

**Authors:** Michael Willmann, Jens P. Bankstahl, Frank M. Bengel, Christine Drechsler, Christina Esdar, Kathrin Gläser, Andrea U. Lopez, Tatjana Ross, Hanno Schieferstein, Tobias L. Ross

**Affiliations:** 1 Department of Nuclear Medicine, Hannover Medical School, Germany.; 2 Merck Healthcare KGaA, Darmstadt, Germany.

**Keywords:** KRAS-G12C, NSCLC, CRC, PET imaging, fluorine-18

## Abstract

Rationale: Mutations in the KRAS gene are some of the most frequent drivers in cancer, including non-small cell lung and colorectal cancer. Covalent inhibitors such as adagrasib, targeting the inactive, GDP-bound form of KRAS-G12C, have shown clinical efficacy but patient selection still depends on invasive biopsies. This study aimed to develop novel fluorine-18 labeled KRAS-G12C inhibitors for noninvasive positron emission tomography (PET) imaging of KRAS mutation status.

Methods: Three KRAS-G12C inhibitors were synthesized by modifying the adagrasib scaffold to allow for radiofluorination. Compounds were evaluated for target affinity and specificity using pERK inhibition assays in KRAS-G12C positive MiaPaCa-2 cells and KRAS protein binding assays. Finally, *in vivo* PET imaging and biodistribution studies were performed in MiaPaCa-2 xenograft-bearing mice under baseline and blocking conditions to assess tumor uptake, specificity, and tracer pharmacokinetics.

Results: In µPET, the tracers showed distinct tumor uptake and biodistribution profiles. Among them, [^18^F]KRAS490 demonstrated the most promising characteristics, with clear tumor accumulation that was significantly reduced under blocking conditions, indicating specific target binding. In contrast, [^18^F]KRAS8125 and [^18^F]KRAS3776 showed limited or non-replaceable tumor uptake. The tracers showed varied clearance patterns, with [^18^F]KRAS490 showing combined hepatobiliary and renal clearance. Notably, [^18^F]KRAS490 also entered the brain, suggesting the potential for central nervous system imaging.

Conclusions: [^18^F]KRAS490 showed specific, blockable tumor uptake and favorable pharmacokinetics, making it a promising tracer for noninvasive imaging of KRAS-G12C mutant tumors. Its ability to penetrate the CNS supports potential application in imaging both peripheral and brain lesions.

## Introduction

Mutations in the KRAS (Kirsten rat sarcoma) gene are found in up to 23% of all human tumors 1, making KRAS one of the most common oncogenic drivers in cancer. These single-base missense mutations are most frequently observed at codon 12 (G12), codon 13 (G13) or codon 61 (Q61) 2 and are especially prevalent in pancreatic adenocarcinoma (PDAC), colorectal adenocarcinoma (CRC) and non-small-cell lung carcinoma (NSCLC) 3. Among these, the KRAS-G12C mutation is dominant in NSCLC (approximately 12%) and occurs to a lesser extent in CRC (3%) and PDAC (1%) 4.

KRAS functions as a molecular switch between active (guanosine triphosphate (GTP) -bound) and inactive (guanosine diphosphate (GDP) -bound) state, regulated by upstream signaling and GTPase-activating proteins (GAPs). Mutations such as G12C impair GAP-mediated GTP hydrolysis, shifting the balance toward the active state and driving activation of persistent downstream signaling cascades such as RAF-MEK-ERK. This sustained signaling promotes proliferation and survival, contributing to oncogenesis **5-7**.

The development of covalent KRAS-G12C inhibitors such as sotorasib and adagrasib has set a major milestone in targeted cancer therapy. These inhibitors bind selectively to the G12C-cysteine residue in the GDP-bound conformation of KRAS, locking it in its inactive state and therefore inhibiting oncogenic downstream signaling. Both sotorasib and adagrasib have received approval for the treatment of metastatic, KRAS-G12C positive NSCLC **2**.

Despite this clinical progress, identifying patients who may benefit from KRAS-G12C targeted therapies remains challenging. Current clinical practice relies on polymerase chain reaction (PCR)- or next generation sequencing (NGS)-based genotyping of tumor biopsies **8**, which is limited by availability of biopsies, tumor heterogeneity and correct localization. A noninvasive, whole-body imaging approach would provide substantial clinical value to patient selection and dynamic monitoring of treatment response and tumor progression.

To address this unmet demand, we designed a series of fluorine-18 labeled KRAS-G12C inhibitors for positron emission tomography (PET) imaging. Building on the core pharmacophore of adagrasib, we modified the pyrrolidine ring to incorporate ^18^F-labeled ethylene and oligoethylene glycol linkers. This design aimed to preserve target binding while enabling ^18^F-labeling for *in vivo* imaging. Here, we present the (radio-)synthesis, *in vitro* characterization and preclinical PET evaluation of three novel KRAS-G12C targeted tracers.

## Methods

### Chemistry

Reference compounds were either purchased or synthesized according to literature procedures. All precursors and cold reference compounds mentioned have been synthesized for the work presented here. A detailed description of the syntheses can be found in the supplementary materials.

### Animals

Male NOD.CB17-*Prkdc^scid^* mice, aged 8-10 w, were obtained from Charles River (Sulzfeld, Germany) and housed in groups under a 14-h light/10-h dark cycle with nesting material, standard laboratory diet and ad libitum access to water. All animal experiments were approved by the local state authority (Landesamt für Verbraucherschutz und Lebensmittelsicherheit (LAVES); ID: 33.12-42502-04-19/3264) and conducted in accordance with European and international guidelines.

### Tumor Inoculation

MiaPaCa-2 cells (ATCC No. CRL-1420) were grown at 37 °C (humidified incubator with 5% CO_2_) in Dulbecco's Modified Eagle's Medium (DMEM) containing fetal bovine serum (FBS) in a final concentration of 10% and horse serum in a final concentration of 2.5%. Cells were harvested for tumor inoculation at 70-80% confluency. The cells were suspended in 1.0 mL phosphate buffered saline (PBS) in a concentration of 67 million cells per millilitre. A 75 µL aliquot, containing 5 million cells, was injected subcutaneously into both flanks of the animal. The mice were controlled in a 2 days interval for tumor growth and health status. Tumors were allowed to grow until they reached an average diameter of 2-5 mm.

### Small Animal Imaging

Mice underwent PET imaging using a rodent imaging chamber (Minerve) in a dedicated small animal PET/CT system (Inveon, Siemens) under isoflurane anesthesia (1.5-2.0% in 0.6 L/min oxygen). Respiration rates were continuously monitored. Images were reconstructed using the maximum a posteriori (MAP) algorithm with 18 iterations, generating a 128 × 128 × 159 matrix. Reconstruction was based on three-dimensional ordered subset expectation maximization (OSEM3D) with 2 iterations. To provide anatomical correlation, a low dose computed tomography (CT) scan was acquired (50 kV, 500 µA, 300 ms, 360 projections), following the PET scan.

For [^18^F]KRAS PET scans, radioactive probes (10.9 ± 1.4 MBq) were administered as a 100 µL bolus via catheter inserted into a lateral tail vein and flushed with 50 µL heparinized saline. For blocking studies, adagrasib or 1-((2*R*,5*S*)-4-((*R*)-6-chloro-2-(3-(dimethylamino)azetidin-1-yl)-8-fluoro-7-(6-methyl-1*H*-indazol-7-yl)quinazolin-4-yl)-2,5-dimethylpiperazin-1-yl)prop-2-en-1-one (KRAS3223, patent: WO17087528) (0.5 µmol in 75 µL), as indicated, was administered 15 min before the respective [^18^F]KRAS tracer, followed by a 50 µL flush of heparinized saline. For tumor localization, a [^18^F]FDG PET scan was subsequently conducted. [^18^F]FDG (11.2 ± 2.5 MBq) was administered as a 100 µL bolus via a intraperitoneally inserted catheter, followed by a 50 µL flush of heparinized saline. After 20 min uptake of [^18^F]FDG under anesthesia, a static 10 min acquisition was performed. Animals remained under anesthesia and were euthanized by cervical dislocation without regaining consciousness.

For analysis, a 10 min frame (50-60 min after tracer injection, [^18^F]KRAS3776, [^18^F]KRAS8125, [^18^F]KRAS490) or a 10 min scan ([^18^F]FDG) was evaluated using PMOD 4.3 (PMOD Technologies LLC, Bruker Preclinical Imaging Division). 60 min time activity curves were additionally visualized. Results were expressed as percent injected dose per gram tissue (% ID/g). A standardized volume of interest (VOI) was placed in the tumor or organ of interest using the CT scan for anatomical guidance.

### Radiolabeling

[^18^F]Fluoride was generated by proton irradiation of enriched [^18^O]H_2_O (11 MeV) using an Eclipse HP cyclotron (Siemens AG) via the ^18^O(p,n)^18^F nuclear reaction. Subsequent synthesis of [^18^F]FDG was performed on a FASTlab™ automated module (GE Healthcare, UK) utilizing a GMP-compliant single-use cassette system. The final product was formulated for routine clinical use in-house.

**[^18^F]KRAS8125 and [^18^F]KRAS490:** [^18^F]Fluoride (50-80 GBq) was eluted from a QMA carbonate cartridge (Waters™, Germany) with a solution of Et_4_NHCO_3_ (2.6 mg) in MeOH (1.3 mL). The solvent was removed at 100 °C in vacuo under a stream of argon and cooled down before a solution of the respective precursor carrying a toluene sulfonyl leaving group (4.0 μmol) in 1.0 mL of dry acetonitrile (MeCN) was added and the reaction mixture was subsequently heated at 70 °C for 20 min. The solution was diluted with 1 mL MeCN and passed through a preconditioned (MeCN) Sep-Pak Silica Plus light cartridge (Waters™, Germany) to remove unreacted [^18^F]fluoride. The products were isolated by semi-preparative HPLC (Phenomenex Luna C18, 250×10 mm; eluent: water/MeCN/ 35:65 (v/v); flow rate: 3 mL/min; t_R_ = 14 min). The collected fraction was diluted 1:2 (v/v) with water before it was passed through a preconditioned (3 ml MeCN, 5 ml water) Sep-Pak C18 Plus Light cartridge (Waters™, Germany). The cartridge was washed with water (3 mL) and eluted with 1 mL ethanol. For *in vitro* and *in vivo* applications, the solvent was removed under reduced pressure at 80 °C and the tracer was subsequently reconstituted in 0.1% polysorbate 80 solution.

**[^18^F]FEtTOs:** [^18^F]Fluoride (30-50 GBq) was eluted from a QMA carbonate cartridge with a solution of KHCO_3_ (2.8 mg) in 0.23 mL H_2_O and K_222_ (8.4 mg) in 1.1 mL MeCN. The solvent was removed azeotropically under reduced pressure at 100 °C assisted by a stream of argon. After cooling down, ethylene ditosylate (10 mg, 27 µmol) in 1.0 mL dry MeCN was added and the reaction mixture was heated for 10 min at 100 °C. The product was isolated by semi-preparative HPLC (Phenomenex Luna C18, 250×10 mm; eluent: water/MeCN/ 50:50 (v/v); flow rate: 3 mL/min; t_R_ = 16 min) and the collected fraction diluted 1:1 (v/v) with water before it was passed through a preconditioned (3 ml MeCN, 5 ml water) Oasis HLB Plus Light Cartridge (Waters™, Germany). The cartridge was washed with water (3 mL) and used in the next step for the synthesis of [^18^F]KRAS3776.

**[^18^F]KRAS3776:** [^18^F]FEtTOs was eluted from the HLB cartridge into a vial containing the precursor KRAS4862 (2.0 mg, 4.3 µmol) using an aqueous solution of KHCO_3_ (1.7 mg in 0.14 mL H_2_O), K222 (5.0 mg) in 0.36 mL MeCN, and 100 µL DMSO. The reaction mixture was heated for 10 min at 100 °C while the pressure was reduced to 400-500 mbar to allow for azeotropic removal of water and acetonitrile. After cooling down, 3 mL MeCN was added and the product was isolated by semi-preparative HPLC (Phenomenex Gemini C18, 250×10 mm; eluent: water/MeCN/ 40:60 (v/v); flow rate: 3 mL/min; tR = 15 min). The collected fraction was diluted 1:1 (v/v) with water before it was passed through a preconditioned (3 ml MeCN, 5 ml water) Sep-Pak C18 Plus Light cartridge (Waters™, Germany). The cartridge was washed with water (3 mL) and eluted with 1 mL ethanol. For *in vitro* and *in vivo* applications, the solvent was removed under reduced pressure at 80 °C and the tracer was subsequently reconstituted in 0.1% polysorbate 80 solution.

### Cellular pERK1/2 Assay

NCI-H358 (ATCC No. CRL-5807) cells were seeded in RPMI cell culture medium supplemented with 10% FBS, 1 mM L-glutamine and 1 mM sodium pyruvate in 96-well plates with 2.5 x 10^4^ cells per well. The following day, cells reached about 90% confluence and were treated with 1:5 serial dilutions of test compounds ranging from 10 µM to 0.65 nM or DMSO in triplicates for 6 h. Cells were washed once with Dulbecco's Phosphate Buffered Saline (DPBS) and lysed with 50 µL ice-cold lysis buffer (RIPA-C lysis buffer from US Biological (Cat. #R2031-75) supplemented with 1 mM sodium orthovanadate (BioLabs, Cat. #P0758S), 1% Phosphatase Inhibitor Cocktail Set II (Calbiochem, Cat. #524625), 0.2% Protease Inhibitor Cocktail Set III (Calbiochem, Cat. #539134) and 0.1% benzonase (Novagen, Cat. #70664). Cellular levels of ERK1/2 phosphorylation were detected using the MSD MULTISPOT Biomarker Detection Base Kit Phospho (T202/Y204; T185/Y187) / Total ERK1/2 according to the manufacturer’s instruction (MSD, #K15107A-3). Counts from DMSO treated control cells were defined as maximum signal (100%) and compound treated samples were calculated as percent of control. Fitting of dose response curves and calculation of IC_50_ values were performed with nonlinear least squares regression analysis using GraphPad Prism software (version 8).

### KRAS Protein Binding Assay

The protein binding assay was conducted following previously established protocols 9,10. Probes were dissolved in a buffer composed of 10% DMSO, 5 mM magnesium chloride (MgCl₂), and 5 mM GDP with an activity of 37 MBq/mL. KRAS protein (either G12C, G12D or Q61H as specified) was incubated at a final concentration of 2 µM with 3.7 MBq of the probes in a buffer containing 20 mM HEPES at pH 7.5, 150 mM sodium chloride (NaCl), 1 mM MgCl₂, and 1 mM dithiothreitol (DTT) for 1 h. The reaction was terminated by the addition of formic acid to a final concentration of 0.2%. The percentage of protein-bound probes was determined as the protein binding rate (%) based on radio-thin layer chromatography (radio-TLC) results.

### Statistical Analysis

All data are expressed as mean ± standard deviation unless stated otherwise. For experiments with n = 2, individual values are reported. Statistical analyses were performed using GraphPad Prism version 8 or 10. Normality of data distribution was assessed prior to further analysis. For dynamic PET/CT measurements data, one-way analysis of variance (ANOVA) was applied, followed by Dunnett’s multiple comparisons test. Where applicable, two-tailed Student’s t-tests were used. A p-value of less than 0.05 was considered statistically significant.

## Results

### Radiochemistry

[^18^F]KRAS8125 and [^18^F]KRAS490 were synthesized via late-stage radiofluorination, wherein the tosylate leaving group of the respective precursors was substituted with fluorine-18. First attempts to label [^18^F]KRAS490 by utilizing the Kryptofix 2.2.2.-potassium complex (K.2.2.2./K⁺) failed. Alternative approaches using tetrabutyl- or tetraethylammonium bicarbonate as bases and counterpart of the nucleophilic [^18^F]fluoride resulted only in trace amounts of [^18^F]KRAS490 and various radiolabeled byproducts indicating a temperature driven degradation process of the labeled product or the tosylate precursor. Anticipating thermodynamic lability of the compounds involved, optimized reaction conditions by reducing the reaction temperature to 70 °C led to moderate analytical radiochemical yields of 15–17% (Table 1). The chemical identity of the radiolabeled product was confirmed by high-performance liquid chromatography (HPLC) through co-injection with the non- radioactive reference compound.

Subsequently, the optimized reaction conditions (Table 1, entry 6) were transferred to an automated synthesis module for the production of [^18^F]KRAS8125 and [^18^F]KRAS490, using higher starting activities (53 ± 12 GBq). The automated synthesis, which included the processing of aqueous [^18^F]fluoride, followed by reaction and subsequent isolation via semi-preparative high-performance liquid chromatography (HPLC), resulted in radiochemical yields (RCY) of 2.3 ± 1.3% and 2.5 ± 1.7% for [^18^F]KRAS490 and [^18^F]KRAS8125, respectively. The final products exhibited excellent radiochemical purities (RCP) exceeding 99%, with a total synthesis time of 92 ± 16 min. Due to carrier levels being below the limit of detection, the molar activity was determined to be greater than 500 GBq/µmol.

Additionally, a two-step strategy was adopted for the radiosynthesis of [^18^F]KRAS3776. This method uses 2-[^18^F]fluoroethyltosylate ([^18^F]FEtOTs) as a prosthetic group for the indirect labeling of the aromatic hydroxyl on the pyrimidine ring of the precursor KRAS4862. [^18^F]FEtOTs was synthesized in an automated synthesis according to literature 11 and obtained with a radiochemical yield of 46 ± 7% and high radiochemical purity of >99% within 84 ± 7 min.

Different reaction parameters were systematically optimized for the (radio)fluoroalkylation reaction as summarized in Table 2. Various base-solvent combinations were tested under variation of reaction temperature and time. Initial attempts using NaOH, TEAHC, or KHCO_3_ alone in dimethylformamide (DMF), dimethyl sulfoxide (DMSO), or MeCN resulted in low radiochemical yields (analytical RCY <2%). The addition of a phase transfer catalyst (K_222_) with KHCO_3_ and iodine in DMSO significantly improved labeling efficiency, resulting in an analytical RCY of 42% (Table 2, entry 11). Further refinement led to optimized conditions using KHCO_3_/K_222_ in DMSO at 80 °C for 10 min, yielding a maximum analytical RCY of 62% (Table 2, entry 12). Notably, the most efficient reaction setup involved eluting [^18^F]FEtOTs directly from the HLB cartridge, where it had been retained in the preceding step, into the reaction vial containing the precursor. This was achieved using the same KHCO_3_/K_222_ eluent used for [^18^F]fluoride elution, added with DMSO. The subsequent reaction is conducted under reduced pressure to enable the azeotropic removal of MeCN and water, leaving DMSO as the only reaction solvent in the vial. With the optimized reaction conditions (Table 2, entry 12) transferred on the automated synthesis module for high starting activities (38.5 ± 2.8 GBq) [^18^F]KRAS3776 was isolated with a RCY of 4.2 ± 1.7% (based on [^18^F]fluoride) and an RCP of >99% within 152 ± 10 min. The carrier content was below the limit of detection, resulting in a molar activity exceeding 500 GBq/µmol. The chemical identity of the radiolabeled product was verified by HPLC through co-injection with the non- radioactive reference compound.

### Cellular pERK Assay

Cellular activity of the three non-radioactive reference probes KRAS490, KRAS8125 and KRAS3776 was investigated in cellular signaling assay in comparison to adagrasib and KRAS3223 which were used for blocking experiments. Detection of P-ERK1/2 inhibition in NCI-H358 cells bearing a heterozygous G12C mutation is a validated downstream marker for KRAS-G12C inhibitors 12.

### KRAS Protein Binding Assay

**Figure 1:**
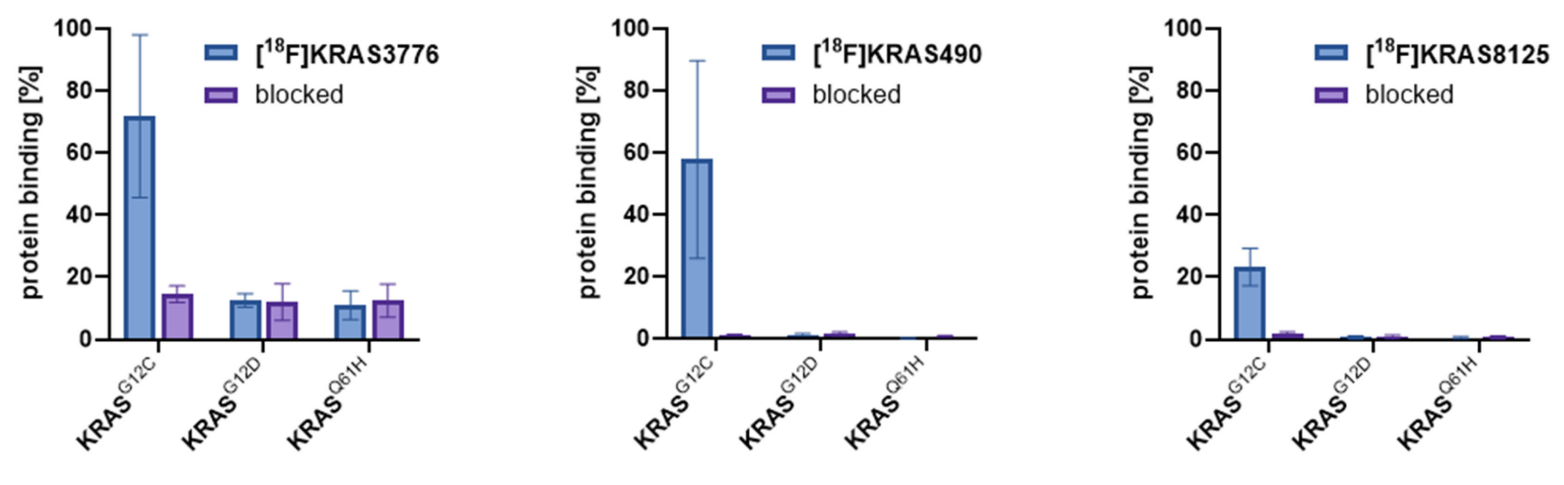
Protein binding assay. The protein binding rates (%, blue bars) of [^18^F]KRAS3776, [^18^F]KRAS490 and [^18^F]KRAS490 were determined as result of radio-TLC (n = 3). Blocking experiments (purple bars) were conducted with adagrasib (0.1 mM). Data are shown as mean ± SD.

The irreversible binding of the probes to the KRAS-G12C protein was tested to check for the specificity of the tracers. Thin-layer chromatography (TLC) enables efficient separation of protein-bound tracer and unbound tracer and can be quantified by radio-thin layer chromatography (radio-TLC). The binding affinities of [^18^F]KRAS8125, [^18^F]KRAS490 and [^18^F]KRAS3776 toward different KRAS variants (G12C, G12D and Q61H) were evaluated using protein-binding assays and results are depicted in Figure 1. [^18^F]KRAS3776 incubated for 60 min showed the highest binding affinity to KRAS-G12C, with a binding rate of 72 ± 21%, while its binding to KRAS-G12D and KRAS-Q61H was significantly lower (12.5 ± 1.8% and 11.0 ± 3.7% respectively). Pre-incubation with adagrasib (0.1 mM) as blocking agent markedly reduced KRAS-G12C binding to 15 ± 2%. In contrast, [^18^F]KRAS490 and [^18^F]KRAS8125 showed lower percentage of binding to KRAS-G12C protein of 58 ± 23% and 23 ± 5% respectively. However, both tracers showed even a negligible binding to the other KRAS mutations KRAS-G12D, KRAS-Q61H as well as a complete blockade of the signal of the KRAS-G12C (<2%) incubation (Figure 1).

### Small Animal PET

To investigate the synthesized and radiolabeled tracers *in vivo* µPET imaging studies of MiaPaCa-2 tumor-bearing mice were conducted to provide information on the biodistribution, especially tumor accumulation, and pharmacokinetics. Tumors were allowed to grow for 3-4 weeks until an average tumor size of 2-5 mm diameter was reached.

**Figure 2:**
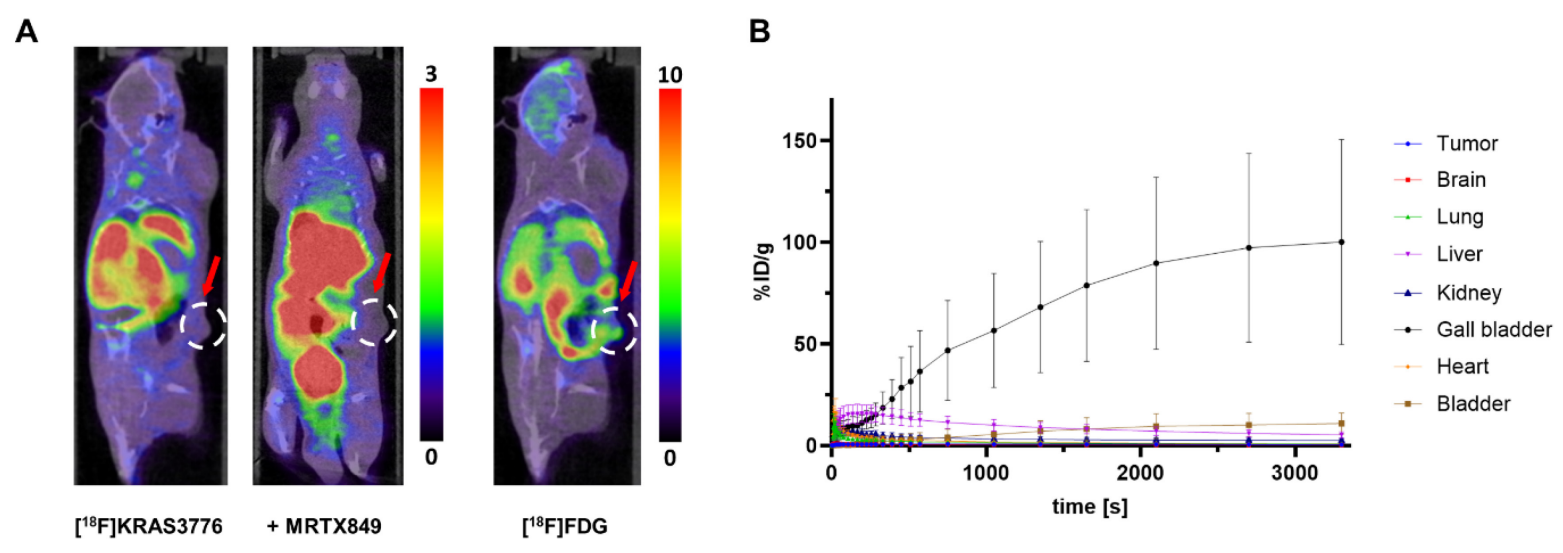
PET imaging and biodistribution of [^18F^]KRAS3776 in tumor-bearing mice (n = 6). (A) Representative coronal PET/CT images of mice at the 60 min timepoint injected with [^18^F]KRAS3776 (left), after pre-blocking with adagrasib (middle), and with [^18^F]FDG (right). No uptake of [^18^F]KRAS3776 in tumor regions (red arrows) is detected. Color bars represent the percentage of injected dose per gram of tissue (% ID/g). (B) Time-activity curves showing the % ID/g for various organs over time. The gall bladder shows increasing signal, suggesting fast and predominant hepatobiliary clearance. Data represent mean ± SD.

[^18^F]KRAS3776 did not substantially accumulate in tumors under both baseline and blocking conditions (Figure 2, A). The tracer however, quickly accumulated in the gall bladder (Figure 2, B), followed by excretion into the intestinal tract, suggesting a predominant hepatobiliary clearance.

**Figure 3:**
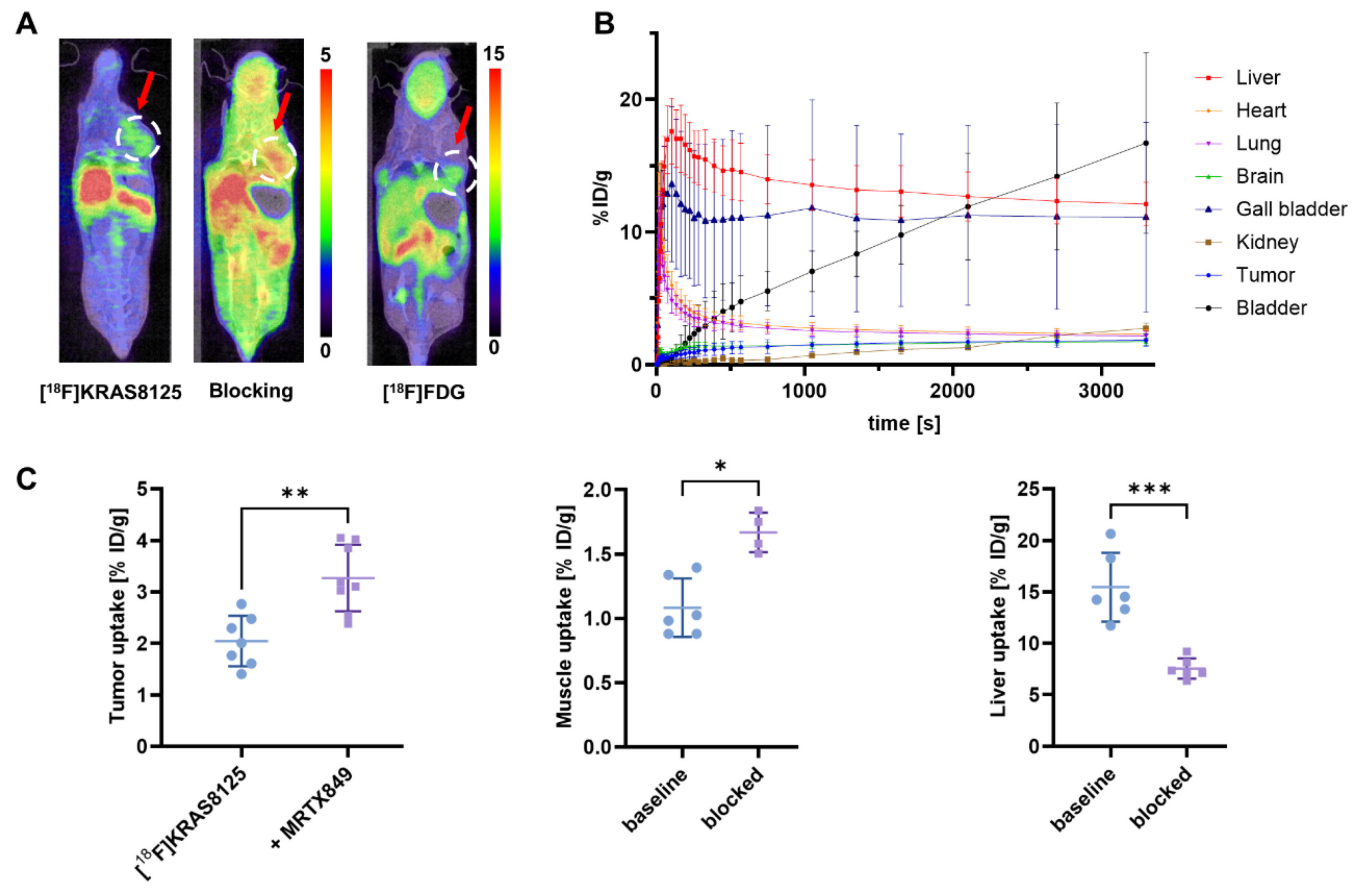
PET imaging and biodistribution of [^18^F]KRAS8125 in tumor-bearing mice (n = 6). (A) Representative coronal PET/CT images of mice at the 60 min timepoint injected with [^18^F]KRAS8125 (left), after pre-blocking with adagrasib (middle), and with [^18^F]FDG (right). Color bars represent the percentage of injected dose per gram of tissue (% ID/g). (B) Time-activity curves showing the % ID/g for various organs over time. Liver, gall bladder and bladder show increasing signal, suggesting hepatobiliary and renal clearance. (C) Tumor- liver- and muscle uptake (% ID/g) under baseline and blocking conditions. Data represent mean ± SD. ***P ≤ 0.001, **P ≤ 0.01, *P ≤ 0.05.

When the PEGylated [^18^F]KRAS8125 was injected and imaged in the same xenograft model, it exhibited considerable uptake (2.05 ± 0.49% ID/g) in the tumor region, suggesting uptake of the tracer in the tumor (Figure 3, A). In a blocking experiment, however, pre-blocking with 0.5 µmol of the KRAS-G12C inhibitor adagrasib 15 min before the scan did not result in a reduction of tumor uptake, indicating that [^18^F]KRAS8125 binding is not effectively blocked under these conditions. Pharmacokinetic analysis indicated a redistribution of tracer activity under blocking conditions where e.g. liver uptake of [^18^F]KRAS8125 was significantly reduced by the presence of adagrasib (Figure 3, C). Alongside, increased levels of circulating radiotracer were observed as evidenced by elevated muscle uptake. Time-activity curves from the baseline PET scans with [^18^F]KRAS8125 (Figure 3, B) revealed a clearance pattern distinct from [^18^F]KRAS3776 with a combination of hepatobiliary and renal excretion.

**Figure 4:**
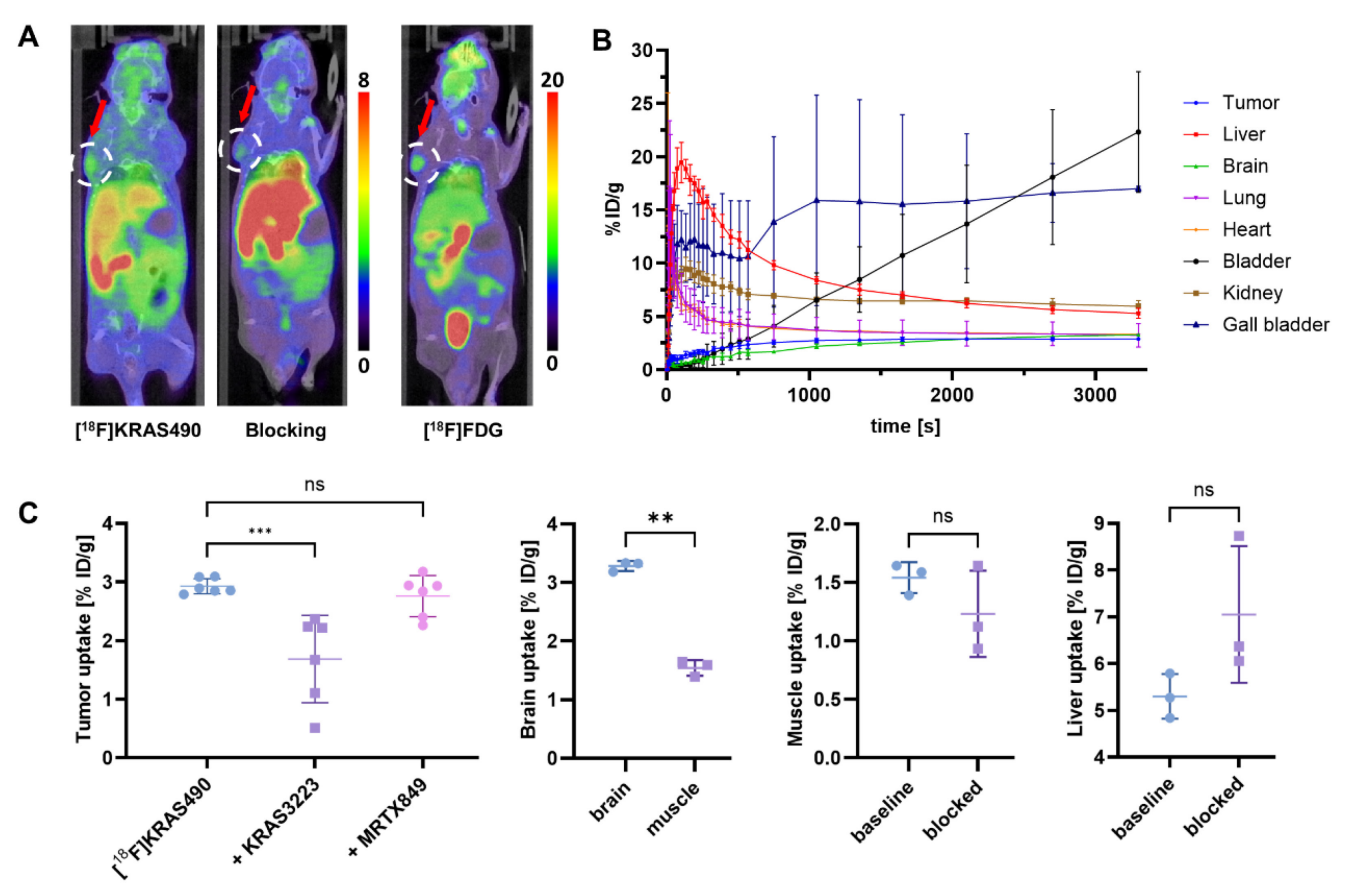
PET imaging and biodistribution of [^18^F]KRAS490 in tumor-bearing mice (n = 3). (A) Representative coronal PET/CT images of mice at the 60 min timepoint injected with [^18^F]KRAS490 (left), after pre-blocking with KRAS3223 (middle), and with [^18^F]FDG (right). Color bars represent the percentage of injected dose per gram of tissue (% ID/g). (B) Time-activity curves showing the % ID/g for various organs over time. Liver, gall bladder and bladder show increasing signal, suggesting hepatobiliary and renal clearance. (C) Tumor-, liver- and muscle uptake (% ID/g) under baseline and blocking conditions and brain- and muscle uptake under baseline conditions. Data represent mean ± SD. ***P ≤ 0.001, **P ≤ 0.01; n.s., not significant.

To optimize blocking conditions, we added the KRAS-G12C inhibitor KRAS3223 (WO17087528) to our blocking experiments in the µPET studies with [^18^F]KRAS490. This tracer showed enhanced tumor uptake (2.93 ± 0.13% ID/g), which was significantly reduced under pre-blocking with 0.5 µmol KRAS3223 (Figure 4, A), indicating a specific blockade of target binding. Notably, liver and muscle uptake remained unchanged under these blocking conditions (Figure 4, C). Interestingly, brain uptake (3.28 ± 0.08% ID/g) was significantly higher than muscle uptake (1.43 ± 0.14% ID/g), suggesting that [^18^F]KRAS490 is able to cross the blood-brain barrier to a certain extent. Time-activity curves revealed a clearance profile similar to that of [^18^F]KRAS8125, characterized by a combination of hepatobiliary and renal excretion (Figure 4, B).

Comparison of the biodistribution profiles (Table 4) revealed pronounced differences among the three tracers. [^18^F]KRAS490 showed the highest overall tissue uptake, including tumor (2.92 ± 0.11% ID/g) and brain (3.28 ± 0.08% ID/g), reflecting improved systemic availability and effective tissue penetration. The comparably high heart uptake (3.31 ± 0.02% ID/g) indicates that [^18^F]KRAS490 remains largely in the circulating blood pool and is not essentially bound to plasma proteins. In contrast, [^18^F]KRAS8125 displayed noticeable higher liver uptake (12.10 ± 1.65% ID/g) and lower activity in heart and tumor, consistent with enhanced hepatic clearance and reduced systemic bioavailability. [^18^F]KRAS3776 exhibited the lowest tissue uptake overall, with particularly high activity in the gall bladder, confirming predominant hepatobiliary excretion.

## Discussion

**Figure 5:**
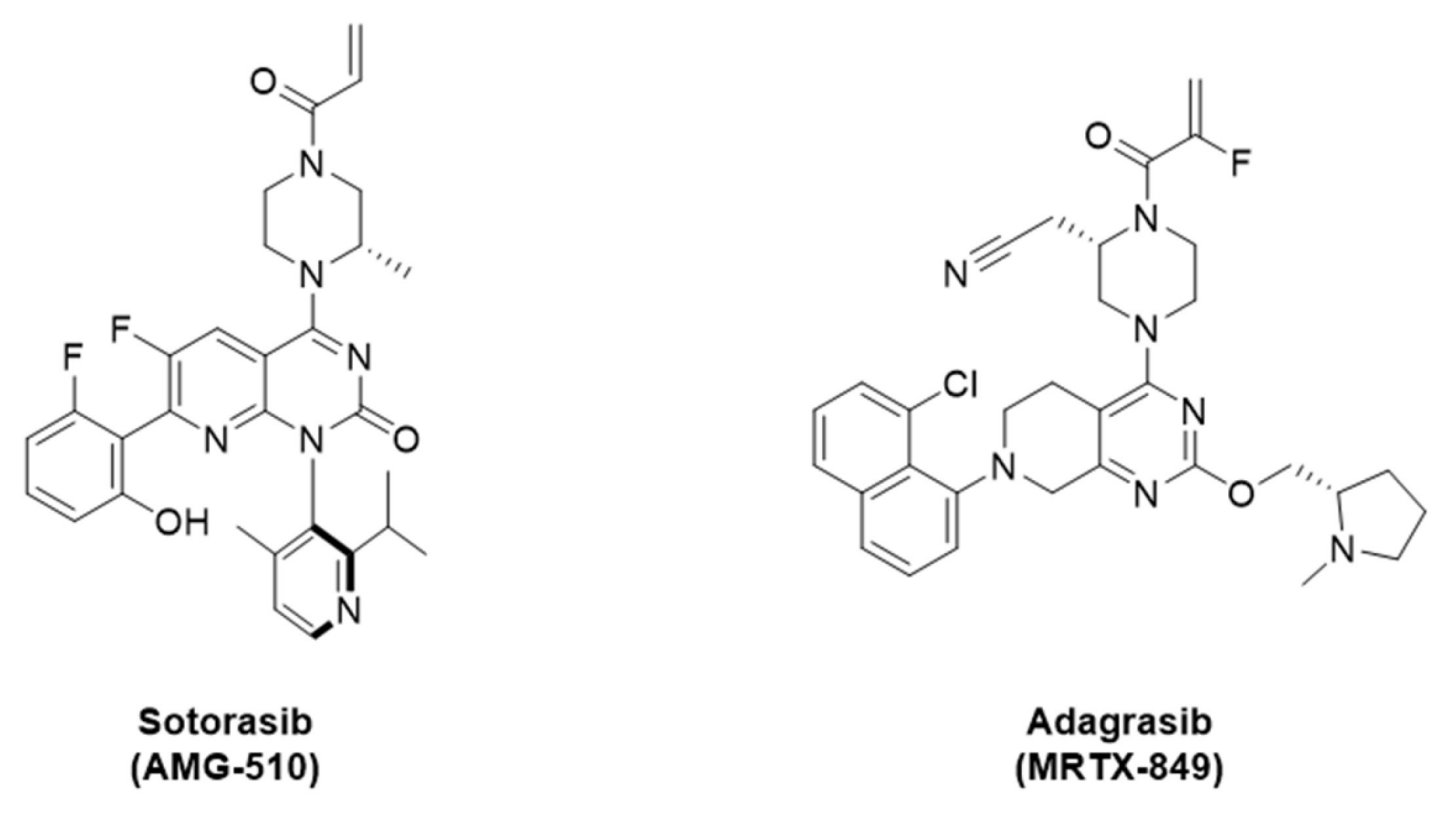
Structures of sotorasib (AMG-510) and adagrasib (MRTX849).

In this study, we successfully synthesized and evaluated three novel KRAS-G12C targeting PET tracers [^18^F]KRAS8125, [^18^F]KRAS490, and [^18^F]KRAS3776. The rationale of our tracer design was built on the structure of the clinically validated KRAS-G12C inhibitor adagrasib (MRTX849), which was identified in previous studies through a series of fragment screens based on a core structure similar to sotorasib (AMG-510, Figure 5). Their structure-based refinement revealed a favorable whole-blood stability of the 2-fluoro acrylamide moiety over the unsubstituted acrylamide, although this modification resulted in a modest reduction of inhibition potency 2.

**Figure 6:**
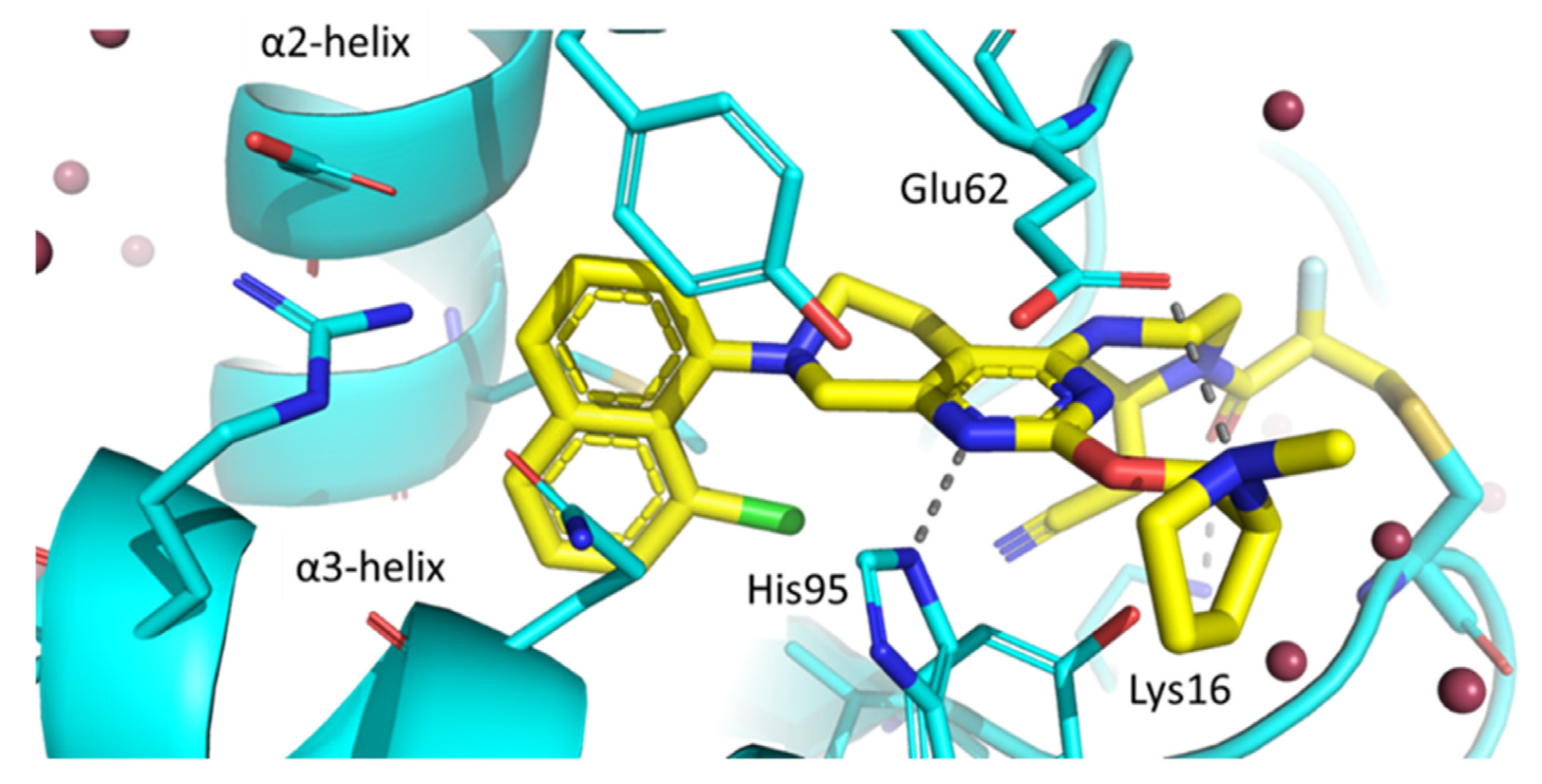
Crystal structure shows the allosteric pocket of KRAS-G12C (blue) bound to adagrasib (yellow) (PDB code 6UT0). The amino acids surrounding the binding site and involved in key hydrogen bonding interactions are shown as sticks **13**.

X-ray crystal structure exposed that adagrasib covalently binds to Cys12 in GDP-bound KRAS-G12C, locking the protein in its inactive state (Figure 6). The naphthalene ring extends into a hydrophobic pocket located between α2 and α3 helices, where it forms van der Waals interactions. Key hydrogen bonds are formed between Lys16 and the acrylamide carbonyl as well as His95 and the pyrimidine nitrogen. Finally, the pyrrolidine ring points towards the solvent forming a salt bridge with the carboxylate group of Glu62 13.

**Figure 7:**
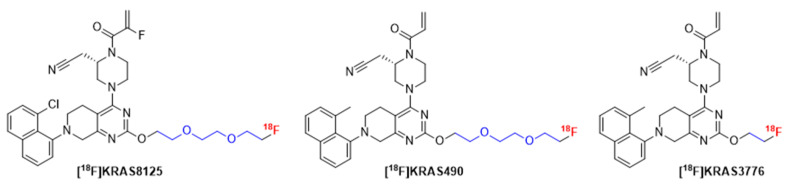
Chemical structure of novel ^18^F-labeled KRAS-G12C inhibitors [^18^F]KRAS8125, [^18^F]KRAS490 and [^18^F]KRAS3776 derived from adagrasib. Modifications were introduced by replacing the pyrrolidine moiety (highlighted in blue) to incorporate ^18^F-labeled linkers suitable for PET imaging while maintaining the core pharmacophore for KRAS-G12C binding.

Starting from these structural insights of adagrasib, we designed novel radiofluorinated KRAS-G12C inhibitors derived from the adagrasib scaffold. We hypothesized that, given the solvent exposure of both Glu62 and the pyrrolidine ring, the salt bridge interaction might not be as critical for binding and decided to introduce different linkers in that position that would be amenable for ^18^F-labeling. Due to their favorable solubility, we focused our attention on ethylene and oligo ethylene glycol linkers (Figure 7). In addition, we extended our structural scope by an unsubstituted acrylamide moiety and replacement of the 8-chloro substituent on the naphthalene unit with a methyl group.

Our initial labeling strategy focused on an indirect radiofluorination method using [^18^F]FEtTOs as prosthetic group, as this method provided a straightforward route to [^18^F]KRAS3776, enabling its production via an automated two-step radiosynthesis that provided the tracer in moderate radiochemical yields and high radiochemical purity while conserving the binding motif of adagrasib. To explore the tracer’s binding characteristics, we conducted cellular pERK1/2 assays to validate sufficient on-target activity as well as cell permeability. In KRAS protein binding assays specificity for KRAS-G12C was demonstrated while no binding to unrelated KRAS mutants such as G12D and Q61H was observed. Although [^18^F]KRAS3776 demonstrated high affinity for KRAS-G12C, competition studies with the adagrasib (G12C-specific inhibitor), along with moderate binding to G12D and Q61H, indicated that the tracer shows a certain degree of unspecific or non-specific binding which cannot be completely blocked. These findings suggested that the tracer's relatively high lipophilicity might contribute to unintended off-target interactions or that the removal of the pyrrolidine ring decreases its selectivity for the KRAS-G12C mutation. Further investigation via dynamic µPET imaging revealed no significant accumulation of [^18^F]KRAS3776 in the KRAS-G12C positive tumors, pointing to a lack of efficient tumor targeting by either not being able to penetrate the cell or reaching the binding site efficiently. Notably, time-activity curves showed highly increased uptake in the gallbladder, suggesting a dominant and fast hepatobiliary clearance. Taken together, these results support the hypothesis that the physicochemical properties of [^18^F]KRAS3776, particularly its lipophilicity, limit its use in KRAS-G12C-targeted imaging due to non-specific binding (*in vitro*) and rapid clearance *in vivo*.

Based on the gained insights, we decided to reduce lipophilicity by introducing a more polar PEG_3_ linker while retaining the overall structure of KRAS3776 to maintain target affinity. [^18^F]KRAS490 was synthesized via late-stage radiofluorination, and after initial challenges with thermal lability of the precursor, optimized reaction conditions could be established. Although radiochemical yields obtained were low, the radiosynthesis was successfully transferred to an automated synthesis setup, enabling reliable production of the tracer while safely using high starting activities of fluorine-18.

The same radiolabeling strategy was applied to the radiosynthesis of [^18^F]KRAS8125, which features a fluoro-substituted acrylamide and an 8-chloro group on the naphthalene ring, rendering it our closest structural analogue to adagrasib. In the protein binding assays [^18^F]KRAS490 exhibited slightly reduced affinity for KRAS-G12C compared to [^18^F]KRAS3776 but also showed minimal non-specific and off-target binding, indicating improved selectivity as intended. The fluoro-substitution on the acrylamide appeared to reduce KRAS-G12C binding affinity of [^18^F]KRAS8125 in the protein assays, while still conserving its selectivity over the off-target mutations G12D and Q61H.

[^18^F]KRAS8125 initially showed promising tumor uptake; however, pre-blocking with adagrasib did not reduce tumor accumulation, despite significant displacement observed in the protein binding assay. This finding demonstrates that [^18^F]KRAS8125 is capable of specific target engagement under controlled conditions, but that systemic pharmacokinetics and clearance pathways rather than binding affinity likely account for the absence of measurable blocking *in vivo*. Supporting this interpretation, we observed a redistribution of tracer activity under blocking conditions, with significantly increased muscle uptake (as a surrogate for circulating activity) and reduced liver uptake compared to baseline scans. This pattern suggests that adagrasib pre-treatment altered hepatic clearance and systemic tracer handling, thereby limiting effective competition at the tumor site. A similar phenomenon, where pre-blocking increased systemic tracer availability was lately reported by Tokala et al 14. While off-target mechanisms cannot be completely excluded, our KRAS protein assays demonstrated selectivity of [^18^F]KRAS8125 for KRAS-G12C over other KRAS mutants, arguing against major unspecific interactions.

To overcome these limitations and explore more effective blocking conditions, we extended our evaluation to [^18^F]KRAS490 and employed the alternative KRAS-G12C inhibitor KRAS3223. [^18^F]KRAS490 showed improved tumor uptake, which was significantly reduced under blocking with KRAS3223 but not with adagrasib, supporting the assumption of more effective competition *in vivo*. Importantly, liver and muscle uptake remained unchanged under blocking conditions, suggesting a more favorable pharmacokinetic profile with reduced interference from systemic redistribution compared to [^18^F]KRAS8125 blocking with adagrasib. Although our *in vitro* assays indicated similar or slightly lower potency of KRAS3223 compared to adagrasib, subtle pharmacokinetic differences such as binding kinetics, free plasma fraction, or tissue penetration may have contributed to the improved *in vivo* blocking efficiency. A more detailed mechanistic analysis would be required to fully resolve these discrepancies but was beyond the scope of this comparative tracer evaluation.

Importantly, [^18^F]KRAS490 demonstrated moderate brain uptake (3.28 ± 0.08% ID/g at 60 min) in the PET scans, which was significantly reduced under blocking conditions (2.48 ± 0.61% ID/g). The extent of uptake is comparable to values reported for established brain-penetrant PET tracers like [^18^F]F-DPA (1.37-4.83% ID/g), [^11^C]PBR28 (1.89-3.59% ID/g) or [^11^C]HD-800 (1.5-5.0% ID/g) whereas non-penetrant tracers typically remain below 1% ID/g 15,16. These findings indicate that [^18^F]KRAS490 is able to cross the blood-brain barrier and that its cerebral uptake can be reduced by blocking, supporting its potential relevance for imaging KRAS-G12C-positive central nervous system (CNS) lesions. This is particularly relevant as CNS metastases occur in 27-42% of patients with NSCLC that harbor KRAS-G12C mutation 17.

## Conclusion

We report the design, radiosynthesis, and biological evaluation of three novel KRAS-G12C-targeted PET tracers: [^18^F]KRAS3776, [^18^F]KRAS8125, and [^18^F]KRAS490. Inspired by the clinically validated inhibitor adagrasib, we explored systematic scaffold modifications to reduce lipophilicity and enhance target specificity. Initial imaging with [^18^F]KRAS3776 showed strong KRAS-G12C affinity *in vitro* but was limited by non-specific binding and rapid hepatobiliary clearance *in vivo*. Introduction of a PEG_3_ linker in [^18^F]KRAS490 successfully reduced lipophilicity, resulting in a favorable pharmacokinetic profile, improved selectivity and effective tumor targeting as demonstrated by significant tracer displacement in blocking experiments. In contrast, [^18^F]KRAS8125 exhibited promising tumor uptake but failed to demonstrate specific competition under blocking conditions, likely due to altered systemic clearance. Notably, [^18^F]KRAS490 also showed brain uptake, suggesting its potential for imaging KRAS-G12C-positive brain metastases. These findings support further preclinical development of [^18^F]KRAS490 as a promising PET imaging agent for determining KRAS mutation status and therapy monitoring.

## Supplementary Material

Supplementary material includes detailed synthetic procedures, NMR/HRMS data and additional experimental information.

## Figures and Tables

**Table 1:** Pilot experiments – evaluated reaction conditions for the radiosynthesis of [^18^F]KRAS490.

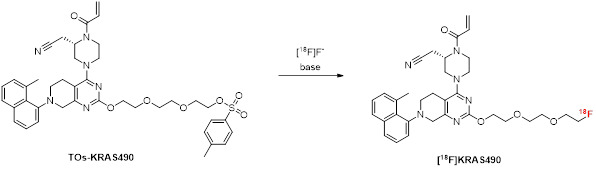				
entry	base	temperature (°C)	time (min)	RCY (%)^a^
1	KHCO_3_/K_222_	100	15	0
2	KHCO_3_/K_222_	70	15	0
3	TBAHCO_3_	100	10	traces
**4**	**TBAHCO_3_**	**70**	**20**	**15**
5	TEAHCO_3_	100	10	traces
**6**	**TEAHCO_3_**	**70**	**20**	**17**

^a^ Determined by radio-HPLC.

**Table 2:** Reaction optimization of the (radio)fluoroalkylation of [¹⁸F]KRAS3776.

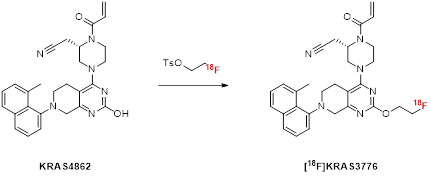					
entry	base	solvent	temperature (°C)	time (min)	RCY (%)^a^
1	NaOH^b^	DMF	100	10	<1
2	NaOH^b^	DMF	80	20	<1
3	NaOH^b^	MeCN	80	10	<1
4	TEAHC^c^	DMF	100	10	<1
5	TEAHC^c^	DMSO	100	10	<1
6	TEAHC^c^	MeCN	80	10	<1
7	KHCO_3_^b^	DMF	100	10	<1
8	KHCO_3_^b^	DMF	80	20	1.2
9	KHCO_3_^b^	DMSO	80	20	1.6
10	KHCO_3_^b^	MeCN	80	20	<1
11^e^	KHCO_3_^c^/K_222_/** I_2_**^d^	DMSO	80	10	42
**12** ^e^	**KHCO_3_** ^c^ **/K_222_** ^c^	**DMSO**	**80**	**10**	**62**
13^e^	KHCO_3_^c^/K_222_^c^	DMSO	80	20	56

^a^Determined by radio-HPLC, ^b^500 µmol, ^c^10 µmol, ^d^traces, ^e^[¹⁸F]FEtOTs is eluted from an HLB cartridge directly into the vial containing the precursor using 0.6 mL of a MeCN/DMSO/H₂O mixture (69:17:14) with the specified base system. The reaction proceeds under reduced pressure to facilitate the removal of MeCN and water.

**Table 3:** Inhibition of P-ERK1/2 signaling in NCI-H358 cells.

	P-ERK1/2 [IC_50_ ± SD (nM); n=2]
KRAS490	173; 183
KRAS8125	895; 931
KRAS3776	571; 489
KRAS3223	29; 42
Adagrasib	25; 31

**Table 4:** Biodistribution of [^18^F]KRAS tracers at 60 min post injection tumor-bearing mice (% ID/g, mean ± SD).

Organs	[^18^F]KRAS3776	[^18^F]KRAS8125	[^18^F]KRAS490
Tumor	0.42 ± 0.24	1.83 ± 0.45	**2.92 ± 0.11**
Brain	0.19 ± 0.03	1.73 ± 0.28	**3.28 ± 0.08**
Heart	0.84 ± 0.29	2.29 ± 0.34	**3.31 ± 0.02**
Liver	5.19 ± 1.59	**12.10 ± 1.65**	5.30 ± 0.48
Gall Bladder	**100.13 ± 50.37**	11.11 ± 7.16	17.01 ± 0.23
Bladder	10.76 ± 5.24	16.71 ± 6.81	**22.33 ± 5.67**
Lung	0.67 ± 0.20	2.16 ± 0.35	**3.23 ± 1.11**
Kidney	2.47 ± 0.88	2.74 ± 0.39	5.97 ± 0.54
Muscle	0.24 ± 0.16	1.02 ± 0.19	1.43 ± 0.14

## Data Availability

The datasets used and/or analyzed during the current study are available from the corresponding author on reasonable request.

## Abbreviations

ANOVA: analysis of variance
CNS: central nervous system
CRC: colorectal carcinoma
CT: computed tomography
DMF: dimethylformamide
DMSO: dimethyl sulfoxide
DTT: dithiothreitol
FBS: fetal bovine serum
FDG: fluorodeoxyglucose
FEtOTs: fluoroethyl tosylate
GAP: GTPase-activating proteins
GDP: guanosine diphosphate
GMP: good manufacturing practice
GTP: guanosine triphosphate
HEPES: 4-(2-hydroxyethyl)-1-piperazineethanesulfonic acid
HPLC: high performance liquid chromatography
ID: injected dose
KRAS: Kirsten rat sarcoma
MeCN: acetonitrile
NaOH: sodium hydroxide
NGS: next generation sequencing
NSCLC: non-small-cell lung carcinoma
PCR: polymerase chain reaction
PDAC: pancreatic ductal adenocarcinoma
pERK: phosphorylated extracellular signal-regulated kinase
PET: positron emission tomography
RCP: radiochemical purity
RCY: radiochemical yield
SD: standard deviation
TEAHC: tetraethylammonium bicarbonate
