# Supplementary material for: Development and in vivo evaluation of 18F-labeled PET tracers covalently targeting KRAS-G12C for noninvasive cancer diagnosis and therapy monitoring
Source: Theranostics. 2026 May 1;16(12):6485–96. doi: 10.7150/thno.123741 (PMC13231836; doi:10.7150/thno.123741)
Supplement: Supplementary file 1 — Supplementary material includes detailed synthetic procedures, NMR/HRMS data and additional experimental information. [file thnov16p6485s1.pdf]

Abbreviations:

AcOH (acetic acid),

ACN (acetonitrile),

BINAP (2,2’-bis(disphenylphosphino)-1,1’-binaphthalene),

dba (dibenzylidene acetone),

tBu (tert-Butyl),

tBuOK (potassium tert-butoxide),

CDI (1,1’-carbonyldiimidazole),

DBU (1,8-diazabicyclo[5.4.0]undec-7-ene),

DCC (dicyclohexylcarbodiimide),

DCM (dichloromethane),

DIAD (diisobutylazodicarboxylate),

DIC (diisopropylcarbodiimide),

DIPEA (di-isopropyl ethylamine),

DMA (dimethyl acetamide),

DMAP (4-dimethylaminopyridine),

DMSO (dimethyl sulfoxide),

DMF (N,N-dimethylformamide),

EDC.HCl (1-ethyl-3-(3-dimethylaminopropyl)carbodiimide hydrochloride),

EtOAc or EE or AcOEt (ethyl acetate),

EtOH (ethanol),

FA (formic acid),

cHex (cyclohexane),

HATU (dimethylamino-([1,2,3]triazolo[4,5-b]pyridin-3-yloxy)-methylene]-dimethyl-ammonium hexafluorophosphate),

HOBt (*N*-hydroxybenzotriazole),

HPLC (high performance liquid chromatography),

hr (hour),

MHz (Megahertz),

MeOH (methanol),

min (minute),

mL (milliliter),

mmol (millimole),

mM (millimolar),

mp (melting point),

MS (mass spectrometry),

MW (microwave),

NMM (N-methyl morpholine),
NMR (Nuclear Magnetic Resonance),
NBS (N-bromo succinimide),
PBS (phosphate buffered saline),
PMB (para-methoxybenzyl),
PyBOP (benzotriazol-1-yl-oxytripyrrolidinophosphonium hexafluoro phosphate),
Rt: room temperature,
Rt: retention time,
TBAF (tetra-butylammonium fluoride),
TBTU (N,N,N′,N′-tetramethyl-O-(benzotriazol-1-yl)uronium tetrafluoroborate), T3P (propane phosphonic acid anhydride),
TEA (triethyl amine),
TFA (trifluoroacetic acid),
THF (tetrahydrofuran),
PetEther (petroleum ether),
TBME (tert-butyl methyl ether),
TLC (thin layer chromatography),
TMS (trimethylsilyl),
TMSI (trimethylsilyl iodide),
UV (ultraviolet),
Xantphos 4,5-bis(diphenylphosphino)-9,9-dimethylxanthene

Methods:

LC-MS:

Method A: HALO C18 2 µm 3.0-30 mm; flow: 1.5 mL/min; T: 40 °C; buffer A: water + 0.05 % TFA; buffer B: ACN + 0.05 % TFA; gradient: 5 % B – 95 % B in 3.0 min.

Method B: HALO C18 2 µm 3.0-30 mm; flow: 1.5 mL/min; T: 40 °C; buffer A: water + 0.1 % FA; buffer B: Acetonitrile + 0.1 % TFA; gradient: 5 % B – 95 % B in 2.0 min.

Method C: HALO C18 2 µm 3.0-30 mm; flow: 1.5 mL/min; T: 40 °C; buffer A: water + 0.1 % FA; buffer B: Acetonitrile + 0.1 % TFA; gradient: 5 % B – 100 % B in 1.2 min.

Method D: HALO C18 2 µm 3.0-30 mm; flow: 1.5 mL/min; T: 40 °C; buffer A: water + 0.05 % TFA; buffer B: ACN + 0.05 % TFA; gradient: 5 % B – 100 % B in 1.2 min.

Method E: HALO C18 2.0 µm 3.0-30 mm; flow: 1.5 mL/min; T: 40 °C; buffer A: water + 0.05 % TFA; buffer B: ACN + 0.05 % TFA; gradient: 5 % B – 100 % B in 1.2 min.

Method F: HALO C18 2.0 µm 3.0-30 mm; flow: 1.5 mL/min; T: 40 °C; buffer A: water + 0.05 % TFA; buffer B: ACN + 0.05 % TFA; gradient: 5 % B – 100 % B in 2.0 min.

Method G: HALO C18 2.0 µm 3.0-30 mm; flow: 1.5 mL/min; T: 40 °C; buffer A: water + 0.05 % TFA; buffer B: ACN + 0.05 % TFA; gradient: 5 % B – 95 % B in 2.0 min.

Method H: Kinetex XB-C18 2.6 µm 3.0-50 mm; flow: 1.2 mL/min; T: 40 °C; buffer A: Water + 0.05 % TFA; buffer B: CAN + 0.05 %TFA; Gradient: 5 % B – 100 % B in 2.0min.

Method I: HALO C18 2 µm 3.0-30 mm; flow: 1.5 mL/min; T: 40 °C; buffer A: water + 0.1 % FA; buffer B: ACN + 0.1 % TFA; gradient: 5 % B – 95 % B in 1.5 min.

Method J: Shim-pack XR-ODS; 2.2 µm, 3.0 x 50 mm; flow: 1.2 mL/min; buffer A: water/0.05 % TFA; buffer B: ACN/0.05 % TFA; gradient: 5 % B – 100 % B in 1.9 min.

Method K: Shim-pack C18; 3.0 µm, 3.0 x 33 mm; flow: 1.5 mL/min; T: 40 °C; buffer A: water + 5mM NH_4HCO_3; buffer B: ACN; gradient: 10 % B – 95 % B in 2.0 min.

Method L: Shim-pack XR-ODS; 2.2 µm, 3.0 x 50 mm; flow: 1.5 mL/min; buffer A: water/0.05 % TFA; buffer B: ACN/0.05 % TFA; gradient: 5 % B – 100 % B in 1.9 min.

Method M: Shim-pack XR-ODS; 2.2 µm, 3.0 x 50 mm; flow: 1.2 mL/min; buffer A: water/0.05 % TFA; buffer B: ACN/0.05 % TFA; gradient: 5 % B – 100 % B in 1.9 min.

Method N: Kinetex C18 2.6 µm 3.0-50 mm; flow: 1.2 mL/min; T: 40 °C; buffer A: Water/5mM NH_4HCO_3; buffer B: ACN; Gradient: 10 % B – 100 % B in 2.0min.

^{1}H-NMR spectra were recorded at a constant temperature of 25 °C on a Varian INOVA 500 spectrometer operating at 499.7 MHz and equipped with a 5 mm ^{1}H{^{13}C-^{15}N} triple resonance Indirect Detection probe or alternatively at a constant temperature of 28 °C on a Varian INOVA 400 spectrometer operating at 400.5 MHz and equipped with a 5 mm ^{1}H{^{15}N-^{31}P} z-axis PFG Indirect Detection probe.

^{13}C-NMR spectra were recorded on a 500 MHz Bruker Avance neo NMR instrument equipped with a broadband cryoprobe.

The HRMS experiments were acquired under full-scan mode over the mass range *m/z* of 100–2000 in positive electrospray ionization (ESI) mode.

An Agilent Infinity II 1290 HPLC system (Agilent Technologies, Santa Clara, United States) coupled to a Fusion Orbitrap mass spectrometer (Thermo Fisher Scientific, Bremen, Germany) was used for the analysis. A YMC-Pack ODS-A column (150 mm × 4.6 mm, 3 µm particle size) (YMC Europe GmbH, Dinslaken, Germany) was applied in gradient elution with the mobile

phase consisting of 0.1% formic acid in water and 0.1% formic acid in acetonitrile. A solution of 0.1mM in acetonitrile/water 50/50 (v/v) was injected at 2 μL.

Intermediates and examples:

Unless otherwise specified, all starting materials are obtained from commercial suppliers and used without further purifications. Unless otherwise specified, all temperatures are expressed in °C and all reactions are conducted at rt. Compounds were purified by common means such as in particular silica chromatography or preparative HPLC.

Piperazine intermediate 1: benzyl (2S)-2-(cyanomethyl)piperazine-1-carboxylate

Benzyl (2S)-2-(cyanomethyl)piperazine-1-carboxylate (Intermediate 1) was synthesized according to known procedures [1].

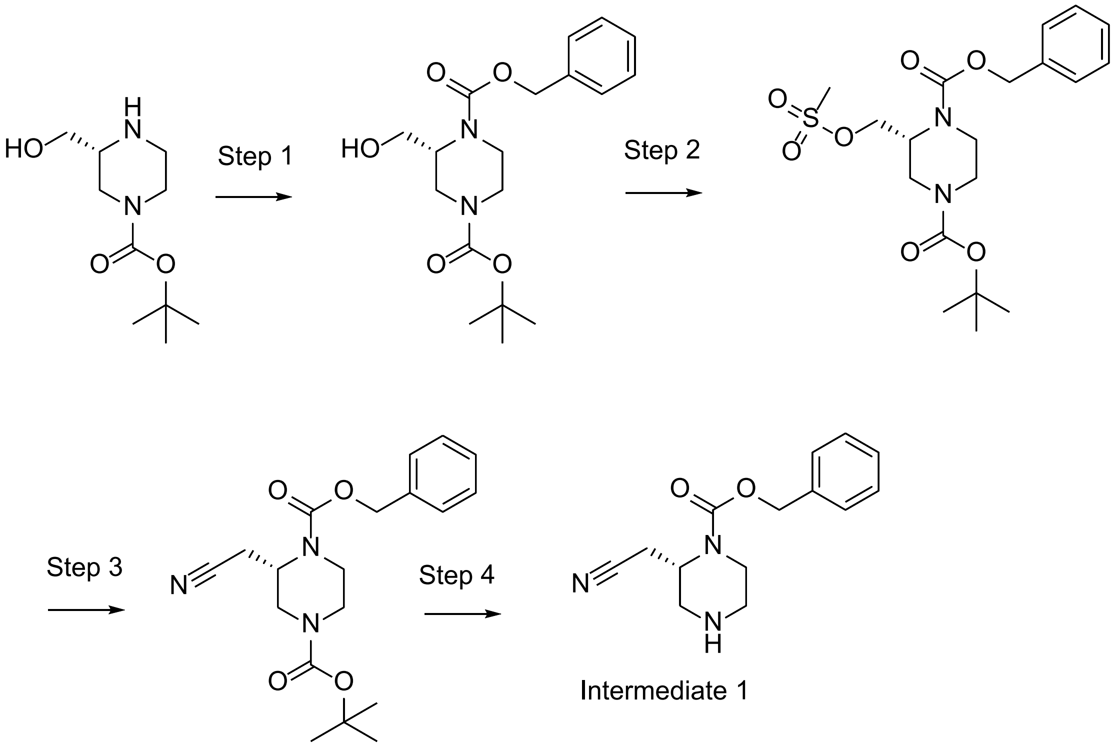

Step 1: 1-benzyl 4-tert-butyl (2R)-2-(hydroxymethyl)piperazine-1,4-dicarboxylate

To a stirred solution of tert-butyl (3R)-3-(hydroxymethyl)piperazine-1-carboxylate (20.00 g; 87.85 mmol; 1.0 eq.) in EtOAc (360 mL) was added an aqueous suspension of $NaHCO_3$ (23.30 g; 263.55 mmol; 3.0 eq.) in H_2O (180 mL). Benzyl chloroformate (Cbz-Cl, 20.51 g; 114.20 mmol; 1.3 eq.) was then introduced dropwise at ambient temperature. The biphasic mixture was stirred overnight. After completion, the layers were separated and the product was isolated by standard aqueous workup followed by drying (Na_2SO_4), filtration, and solvent removal. The crude material was purified by silica gel chromatography (PE/EtOAc, 40:1 → 1:1) to afford 1-benzyl 4-tert-butyl (2R)-2-(hydroxymethyl)piperazine-1,4-dicarboxylate as a light yellow oil (26.0 g; 69.00 mmol; 79% yield; 93% purity).

LC-MS method E: $(M+H)^+$ 351.2; Rt: 0.686 min

Step 2: 1-benzyl 4-tert-butyl (2R)-2-[(methanesulfonyloxy)methyl]piperazine-1,4-dicarboxylate

A solution of 1-benzyl 4-tert-butyl (2R)-2-(hydroxymethyl)piperazine-1,4-dicarboxylate (5.00 g; 13.27 mmol; 1.0 eq.) in THF (60.0 mL) was treated with Et_3N (4.00 g; 39.13 mmol; 3.0 eq.), followed by addition of MsCl (2.30 g; 19.88 mmol; 1.5 eq.) at room temperature. The mixture

was stirred for 1 h and then quenched with water. The product was recovered by extraction with EtOAc (3×); the combined organic phases were processed in the usual manner (brine wash, drying over anhydrous Na_2SO_4, filtration, evaporation) to provide 1-benzyl 4-tert-butyl (2R)-2-[(methanesulfonyloxy)methyl]piperazine-1,4-dicarboxylate as a yellow oil (5.00 g; 11.67 mmol; 88% yield; 100% purity), which was used directly in the next step.

LC-MS method C: $(M+1(-tBu))^+$ 373.0; Rt: 0.734 min

Step 3: 1-benzyl 4-tert-butyl (2S)-2-(cyanomethyl)piperazine-1,4-dicarboxylate

1-benzyl 4-tert-butyl (2R)-2-[(methanesulfonyloxy)methyl]piperazine-1,4-dicarboxylate (40.00 g; 80.93 mmol; 1.0 eq.) was dissolved in DMA (250 mL), and NaCN (16.03 g; 323.73 mmol; 4.0 eq.) was added portionwise. The mixture was heated to 60 °C and stirred for 16 h under nitrogen. After cooling, the reaction mixture was diluted with brine and extracted with EtOAc (4×). The combined extracts were washed with water, dried (anhydrous Na_2SO_4), filtered, and concentrated. Purification by silica gel column chromatography (PE/EtOAc, 4:1) afforded 1-benzyl 4-tert-butyl (2S)-2-(cyanomethyl)piperazine-1,4-dicarboxylate as a yellow oil (25.00 g; 64.97 mmol; 80% yield; 93% purity).

LC-MS method E: $(M-tBu)^+$ 304.2; Rt: 0.763 min

Step 4: benzyl (2S)-2-(cyanomethyl)piperazine-1-carboxylate

1-benzyl 4-tert-butyl (2S)-2-(cyanomethyl)piperazine-1,4-dicarboxylate (25.00 g; 64.97 mmol; 1.0 eq.) was dissolved in dioxane (100 mL). HCl in 1,4-dioxane (12%, 133 mL; 8.1 eq.) was added and the mixture was stirred at room temperature for 3 h. The reaction was neutralized to approximately pH 8 using saturated aqueous $NaHCO_3$ and concentrated to remove volatiles. The residue was partitioned between water and EtOAc; the organic phase was collected after extraction (EtOAc, 3×) and worked up by washing, drying (anhydrous Na_2SO_4), filtration, and evaporation. This afforded benzyl (2S)-2-(cyanomethyl)piperazine-1-carboxylate as a yellow oil (15.00 g; 56.34 mmol; 87% yield; 97% purity), which was used without further purification.

LC-MS method H: $(M+H)^+$ 260.2; Rt: 0.732 min

Intermediate 2: 1-bromo-8-methylnaphthalene

1-bromo-8-methylnaphthalene (Intermediate 2) was synthesized according to known procedures [1].

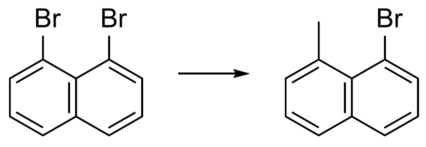

Under an inert atmosphere, 1,8-dibromonaphthalene (50.0 g; 166.10 mmol; 1.0 eq.) was dissolved in dry THF (1.0 L) and cooled to 0 °C. MeLi (1.6 M, 109.0 mL; 174.41 mmol; 1.1 eq.) was added dropwise while maintaining the temperature at 0 °C, and the mixture was stirred for 30 min. Iodomethane (CH_3I, 43.8 mL; 308.28 mmol; 4.0 eq.) was then added dropwise at 0 °C. The reaction was allowed to warm to room temperature and stirred for 3 h. The mixture was cooled to 0 °C and quenched by careful addition of ice/brine. The aqueous layer was separated and extracted with EtOAc (3×). The combined organic phases were dried over Na_2SO_4, filtered, and concentrated under reduced pressure. The residue was purified by silica gel column chromatography (PE/EtOAc, 1:1) to afford crude 1-bromo-8-methylnaphthalene (30.0 g; 88% purity) as a white solid. Recrystallization from i-PrOH (60 mL) provided 1-bromo-8-methylnaphthalene (20.0 g; 85.94 mmol; 52% yield; 95% purity) as a white solid.

^1H NMR (400 MHz, $CDCl_3$-d_6): δ= 7.89 - 7.76 (m, 2H), 7.76 - 7.68 (m, 1H), 7.41 - 7.29 (m, 2H), 7.23 (dd, J = 8.1, 7.4 Hz, 1H), 3.15 (d, J = 1.0 Hz, 3H).

Pyrimidine intermediate 3: benzyl (2S)-4-[2-chloro-7-(8-methylnaphthalen-1-yl)-5H,6H,7H,8H-pyrido[3,4-d]pyrimidin-4-yl]-2-(cyanomethyl)piperazine-1-carboxylate

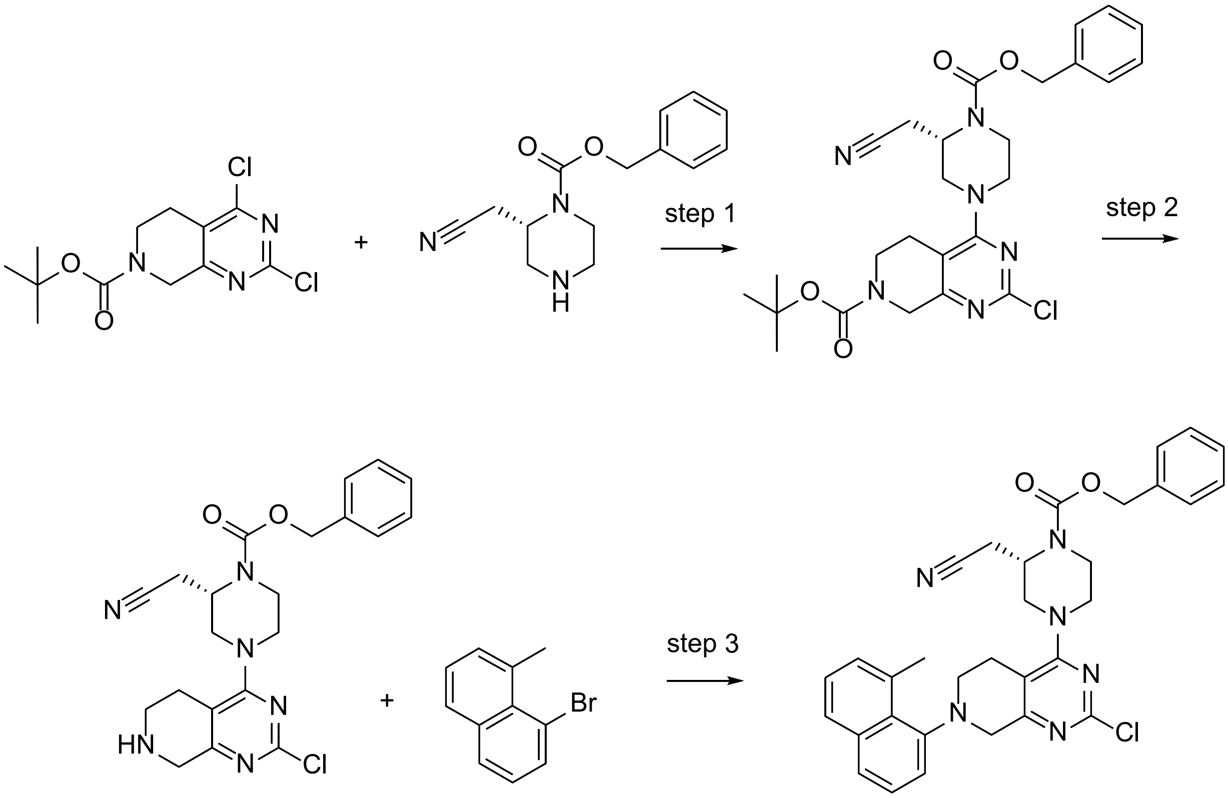

Step 1: benzyl (2S)-4-{7-[(tert-butoxy)carbonyl]-2-chloro-5H,6H,7H,8H-pyrido[3,4-d]pyrimidin-4-yl}-2-(cyanomethyl)piperazine-1-carboxylate

tert-Butyl 2,4-dichloro-5*H*,6*H*,8*H*-pyrido[3,4-d]pyrimidine-7-carboxylate (12.90 g, 41.99 mmol, 1.1 equiv), benzyl (2S)-2-(cyanomethyl)piperazine-1-carboxylate (10.00 g, 38.18 mmol, 1.0

equiv), and DIEA (9.97 g, 76.36 mmol, 2.0 equiv) were combined in DMSO (200 mL) and stirred at 50 °C overnight. After cooling, the mixture was diluted with water and extracted with EtOAc (2×). The combined organic extracts were washed with brine, dried over anhydrous Na_2SO_4, filtered, and concentrated in vacuo. Purification of the crude residue by silica gel chromatography (PE/EtOAc, 2:1) afforded benzyl (2S)-4-[7-(tert-butoxycarbonyl)-2-chloro-5H,6H,8H-pyrido[3,4-d]pyrimidin-4-yl]-2-(cyanomethyl)piperazine-1-carboxylate as a yellow oil (19.0 g, 35.37 mmol, 93% yield, 98% purity).

LC-MS method D: $(M+H)^+$ 527.2; Rt: 0.820 min

Step 2: benzyl (2S)-4-{2-chloro-5H,6H,7H,8H-pyrido[3,4-d]pyrimidin-4-yl}-2-(cyanomethyl)piperazine-1-carboxylate

Benzyl (2S)-4-[7-(tert-butoxycarbonyl)-2-chloro-5H,6H,8H-pyrido[3,4-d]pyrimidin-4-yl]-2-(cyanomethyl)piperazine-1-carboxylate (19.00 g, 35.37 mmol, 1.0 equiv) was dissolved in DCM (380 mL) and cooled to 0 °C. A solution of HCl in 1,4-dioxane (53.0 mL, 209.32 mmol, 5.92 equiv) was added, and the mixture was allowed to warm to room temperature and stirred for 1.5 h. The reaction was then diluted with water and the aqueous phase was adjusted to pH 8 with saturated aqueous $NaHCO_3$ at 0 °C. The mixture was extracted with EtOAc (3×). The combined organic extracts were washed with brine, dried over anhydrous Na_2SO_4, filtered, and concentrated under reduced pressure to afford benzyl (2S)-4-[2-chloro-5H,6H,7H,8H-pyrido[3,4-d]pyrimidin-4-yl]-2-(cyanomethyl)piperazine-1-carboxylate as a yellow solid (15.0 g, 31.66 mmol, 90% yield, 90% purity), which was used directly in the next step.

LC-MS method J: $(M+H)^+$ 427.2; Rt: 0.911 min

Step 3: benzyl (2S)-4-[2-chloro-7-(8-methylnaphthalen-1-yl)-5H,6H,7H,8H-pyrido[3,4-d]pyrimidin-4-yl]-2-(cyanomethyl)piperazine-1-carboxylate

To a stirred solution of benzyl (2S)-4-[2-chloro-5H,6H,7H,8H-pyrido[3,4-d]pyrimidin-4-yl]-2-(cyanomethyl)piperazine-1-carboxylate (6.00 g; 12.66 mmol; 1.0 eq.) and 1-bromo-8-methylnaphthalene(8.84 g; 37.98 mmol; 3.0 eq.) in 1,4-dioxane (122 mL) Cs_2CO_3 (13.03 g, 37.99 mmol, 3.0 eq.), XantPhos (1.54 g, 2.53 mmol, 0.2 eq.) and $Pd_2(dba)_3$ (1.22 g; 1.27 mmol; 0.1 eq.) were added. The resulting mixture was stirred for 12 h at 100 °C under nitrogen. After cooling to room temperature, the reaction mixture was diluted with water and extracted with DCM (3×). The combined organic extracts were washed with water, dried over anhydrous Na_2SO_4, filtered, and concentrated under reduced pressure. The crude residue was purified by silica gel chromatography (PE/EtOAc, 5:1) to give benzyl (2S)-4-[2-chloro-7-(8-methylnaphthalen-1-yl)-5H,6H,8H-pyrido[3,4-d]pyrimidin-4-yl]-2-(cyanomethyl)piperazine-1-carboxylate as a yellow solid (3.00 g, 4.67 mmol, 37% yield, 88% purity).

LC-MS method E: $(M+H)^+$ 567.2; Rt: 0.884 min

Pyrimidine intermediate 4: benzyl (2S)-4-[2-chloro-7-(8-chloronaphthalen-1-yl)-5H,6H,7H,8H-pyrido[3,4-d]pyrimidin-4-yl]-2-(cyanomethyl)piperazine-1-carboxylate

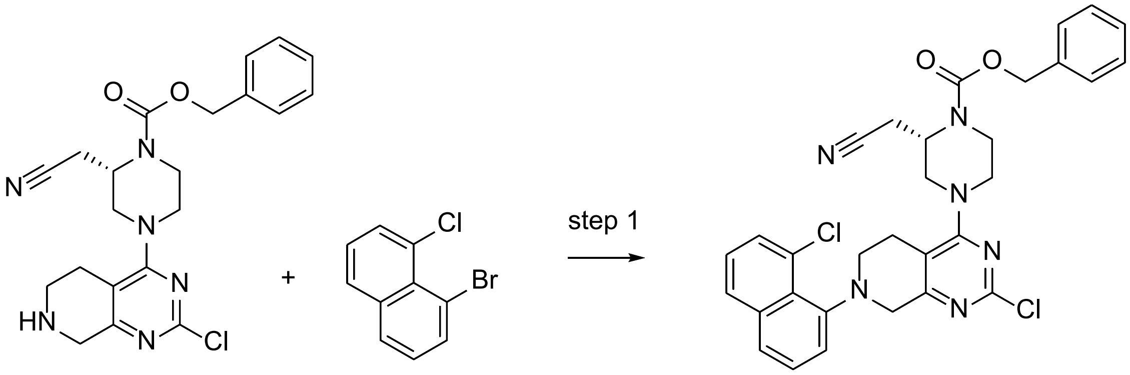

A mixture of benzyl (2S)-4-{2-chloro-5H,6H,7H,8H-pyrido[3,4-d]pyrimidin-4-yl}-2-(cyanomethyl)piperazine-1-carboxylate (24.80 g; 55.65 mmol; 1.0 eq.),1-bromo-8-chloronaphthalene (14.15 g; 55.65 mmol; 1.0 eq.), K_3PO_4 (29.53 g; 139.13 mmol; 2.50 eq.), $Pd_2(dba)_3$ (2.68 g; 2.78 mmol; 0.05 eq.) and XantPhos (3.39 g; 5.57 mmol; 0.10 eq.) in 1,4-dioxane (250 mL) was stirred for 5 h at 100 °C under nitrogen atmosphere. The resulting mixture was cooled to room temperature and concentrated under reduced pressure. It was diluted with water and extracted with EtOAc three times. The combined organic extracts were dried over anhydrous Na_2SO_4, filtered, and concentrated under reduced pressure. The crude material was purified by silica gel chromatography (EtOAc/PE, 1:1) to afford benzyl (2S)-4-[2-chloro-7-(8-chloronaphthalen-1-yl)-5H,6H,7H,8H-pyrido[3,4-d]pyrimidin-4-yl]-2-(cyanomethyl)piperazine-1-carboxylate as a red solid (13.50 g, 22.98 mmol, 41% yield, 100% purity).

LC-MS method B: $(M+H)^+$ 587.1; Rt: 1.292 min

Pyrimidine intermediate 5: tert-butyl (2S)-4-[7-(8-chloronaphthalen-1-yl)-2-methanesulfinyl-5H,6H,7H,8H-pyrido[3,4-d]pyrimidin-4-yl]-2-(cyanomethyl)piperazine-1-carboxylate

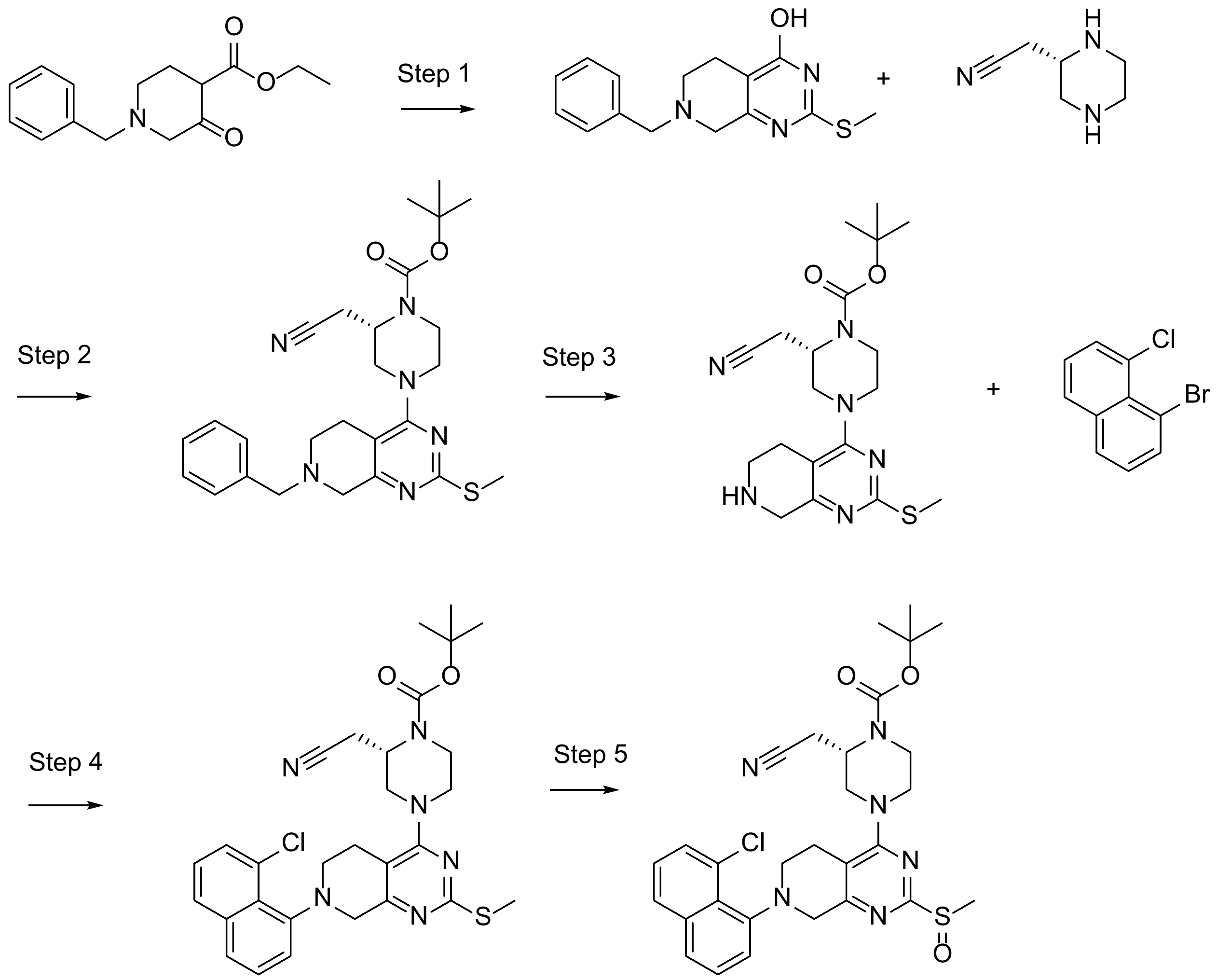

Step 1: 7-benzyl-2-(methylsulfanyl)-5H,6H,7H,8H-pyrido[3,4-d]pyrimidin-4-ol

To a mixture of ethyl 1-benzyl-3-oxopiperidine-4-carboxylate (173.0 mL; 727.09 mmol; 1.0 eq.) and NaOMe (165.38 g; 2908.36 mmol; 4.0 eq.) in MeOH (2000 mL) bis((methylsulfanyl)methanimidamide) sulfuric acid (213.06 g; 727.09 mmol; 1.0 eq.) was added at room temperature. The resulting mxiture was stirred overnight at room temperature.

The mixture was adjusted to pH 7 with using aqueous HCl solution (1M). The precipitated solids were collected by filtration, washed with water and dried over Na_2SO_4 to afford 7-benzyl-2-(methylsulfanyl)-5H,6H,7H,8H-pyrido[3,4-d]pyrimidin-4-ol (149.00 g; 475.20 mmol; 65.4% yield; 92% purity) as an off-white solid. The filtrate was concentrated to a reduced volume until a solid precipitated. The solid was isolated by filtration, washed with water, and dried to give 7-benzyl-2-(methylsulfanyl)-5H,6H,7H,8H-pyrido[3,4-d]pyrimidin-4-ol (8.0 g, 22.63 mmol, 3.1% yield, 81% purity).

LC-MS method B: $(M+H)^+$ 288.2; Rt: 0.511 min

Step 2: tert-butyl (2S)-4-[7-benzyl-2-(methylsulfanyl)-5H,6H,7H,8H-pyrido[3,4-d]pyrimidin-4-yl]-2-(cyanomethyl)piperazine-1-carboxylate

A mixture of 7-benzyl-2-(methylsulfanyl)-5H,6H,7H,8H-pyrido[3,4-d]pyrimidin-4-ol (40.00 g; 116.22 mmol; 1.0 eq.), (benzotriazol-1-yloxy)tris(dimethylamino) phosphonium hexafluorophosphate (62.31 g; 139.47 mmol; 1.2 eq.) and DIEA (121.31 mL; 697.33 mmol; 6.0 eq.) in DMF (400 mL) was stirred for 30 min at room temperature. Then 2-[(2S)-piperazin-2-yl]acetonitrile dihydrochloride (28.19 g; 139.47 mmol; 1.2 eq.) was added. The reaction mixture was stirred at room temperature for 6 h, then cooled to 0 °C and treated with $(Boc)_2O$ (39.3 mL, 174.33 mmol, 1.5 equiv). The mixture was allowed to warm to room temperature and stirred overnight. After dilution with water, the product was extracted with EtOAc (3×). The combined organic layers were dried over anhydrous Na_2SO_4, filtered and concentrated under reduced pressure. The residue was purified by silica gel column chromatography (eluting with 1:1 PE/EtOAc) to afford tert-butyl (2S)-4-[7-benzyl-2-(methylsulfanyl)-5H,6H,7H,8H-pyrido[3,4-d]pyrimidin-4-yl]-2-(cyanomethyl)piperazine-1-carboxylate (30.2 g; 55.49 mmol; 48% yield; 95% purity) as a yellow oil.

LC-MS method F: $(M+H)^+$ 495.25; Rt: 0.871 min

Step 3: tert-butyl (2S)-2-(cyanomethyl)-4-[2-(methylsulfanyl)-5H,6H,7H,8H-pyrido[3,4-d]pyrimidin-4-yl]piperazine-1-carboxylate

tert-Butyl (2S)-4-[7-benzyl-2-(methylsulfanyl)-5H,6H,7H,8H-pyrido[3,4-d]pyrimidin-4-yl]-2-(cyanomethyl)piperazine-1-carboxylate (44.00 g, 84.16 mmol, 1.0 equiv) and DIEA (43.0 mL, 252.47 mmol, 3.0 equiv) were dissolved in DCE (450 mL) and cooled to 0 °C. 1-Chloroethyl carbonochloridate (23.0 mL, 210.39 mmol, 2.5 equiv) was added dropwise and the reaction was stirred at 0 °C for 1 h. Volatiles were removed under reduced pressure and the residue was taken up in MeOH (450 mL). The mixture was heated at 70 °C for 1 h, then cooled to room temperature and washed with petroleum ether. The methanolic phase was concentrated in vacuo and the residue was partitioned between EtOAc and water. The aqueous layer was adjusted to pH ~8 with saturated $NaHCO_3$ solution, separated, and extracted with EtOAc (2×). The combined organic extracts were dried over Na_2SO_4 and concentrated under reduced pressure to afford tert-butyl (2S)-2-(cyanomethyl)-4-[2-(methylsulfanyl)-5H,6H,7H,8H-pyrido[3,4-d]pyrimidin-4-yl]piperazine-1-carboxylate as a brown oil (31.00 g, 64.36 mmol, 77% yield, 84% purity).

LC-MS method B: $(M+H)^+$ 405.20; Rt: 0.683 min

Step 4: tert-butyl (2S)-4-[7-(8-chloronaphthalen-1-yl)-2-(methylsulfanyl)-5H,6H,7H,8H-pyrido[3,4-d]pyrimidin-4-yl]-2-(cyanomethyl)piperazine-1-carboxylate

A mixture of tert-butyl (2S)-2-(cyanomethyl)-4-[2-(methylsulfanyl)-5H,6H,7H,8H-pyrido[3,4-d]pyrimidin-4-yl]piperazine-1-carboxylate (29.00 g; 60.21 mmol; 0.7 eq.), tert-butyl (2S)-2-(cyanomethyl)-4-[2-(methylsulfanyl)-5H,6H,7H,8H-pyrido[3,4-d]pyrimidin-4-yl]piperazine-1-

carboxylate (10.50 g; 24.38 mmol; 0.3 eq.), $Pd_2(dba)_3$ (8.15 g; 8.46 mmol; 0.1 eq.), XantPhos (10.30 g; 16.92 mmol; 0.2 eq.) and K_3PO_4 (56.70 g; 253.77 mmol; 3.0 eq.) in dioxane (400 mL) was stirred overnight at 100 °C under nitrogen atmosphere. The reaction mixture was cooled to room temperature and concentrated under reduced pressure. The resulting mixture was partitioned between EtOAc and water. The organic layer was separated, dried over anhydrous Na_2SO_4, filtered and concentrated under reduced pressure. The residue was purified by silica gel column chromatography, eluted with EtOAc: PE (1:1), to afford tert-butyl (2S)-4-[7-(8-chloronaphthalen-1-yl)-2-(methylsulfanyl)-5H,6H,7H,8H-pyrido[3,4-d]pyrimidin-4-yl]-2-(cyanomethyl)piperazine-1-carboxylate (27.90 g; 45.69 mmol; 64% yield) as a yellow solid.

LC-MS method F: (M+H) 565.35; Rt: 1.054 min

Step 5: tert-butyl (2S)-4-[7-(8-chloronaphthalen-1-yl)-2-methanesulfinyl-5H,6H,7H,8H-pyrido[3,4-d]pyrimidin-4-yl]-2-(cyanomethyl)piperazine-1-carboxylate

A solution of tert-butyl (2S)-4-[7-(8-chloronaphthalen-1-yl)-2-(methylsulfanyl)-5H,6H,7H,8H-pyrido[3,4-d]pyrimidin-4-yl]-2-(cyanomethyl)piperazine-1-carboxylate (8.60 g; 7.82 mmol; 0.2 eq.), tert-butyl (2S)-4-[7-(8-chloronaphthalen-1-yl)-2-(methylsulfanyl)-5H,6H,7H,8H-pyrido[3,4-d]pyrimidin-4-yl]-2-(cyanomethyl) piperazine-1-carboxylate (26.90 g; 44.05 mmol; 0.9 eq.) and 2-(benzenesulfonyl)-3-phenyloxaziridine (17.12 g; 62.24 mmol; 1.2 eq.) in DCM (360 mL) was stirred for 2 h at room temperature. The resulting mixture was concentrated under vacuum. The crude residue was purified by silica gel chromatography (DCM/MeOH, 10:1) and further purified by reverse-phase chromatography (C18 silica gel column; mobile phase A: water (containing 5mM NH_4HCO_3) and B: CH_3CN, gradient: 40% to 80% in 40 min; detector: UV 220/254 nm) to obtain tert-butyl (2S)-4-[7-(8-chloronaphthalen-1-yl)-2-methanesulfinyl-5H,6H,7H,8H-pyrido[3,4-d]pyrimidin-4-yl]-2-(cyanomethyl)piperazine-1-carboxylate (25.3 g; 40.96 mmol; 79% yield) as a yellow oil.

LC-MS method B: $(M+H)^+$ 581.20; Rt: 1.077 min

KRAS490: Example 1: 2-[(2S)-4-(2-{2-[2-(2-fluoroethoxy)ethoxy]ethoxy}-7-(8-methylnaphthalen-1-yl)-5H,6H,7H,8H-pyrido[3,4-d]pyrimidin-4-yl)-1-(prop-2-enoyl)piperazin-2-yl]acetonitrile

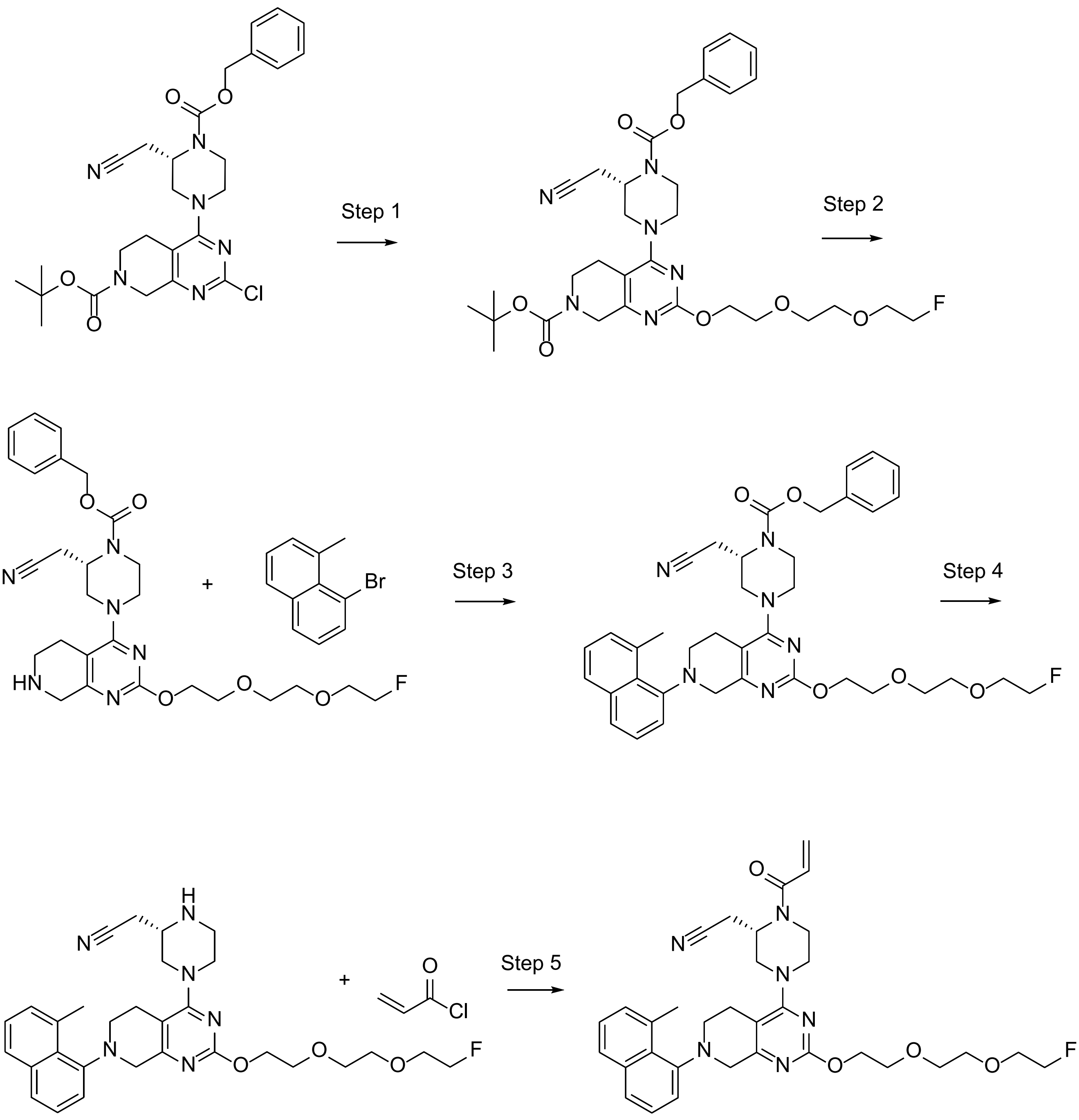

Step 1: benzyl (2S)-4-{7-[(tert-butoxy)carbonyl]-2-{2-[2-(2-fluoroethoxy)ethoxy] ethoxy}-5H,6H,7H,8H-pyrido[3,4-d]pyrimidin-4-yl}-2-(cyanomethyl)piperazine-1-carboxylate

Benzyl (2S)-4-{7-[(tert-butoxy)carbonyl]-2-chloro-5H,6H,7H,8H-pyrido[3,4-d]pyrimidin-4-yl}-2-(cyanomethyl)piperazine-1-carboxylate (200 mg, 0.36 mmol, 1.0 equiv), Cs_2CO_3 (354 mg, 1.08 mmol, 3.0 equiv), RuPhos Palladacycle Gen.3 (15 mg, 0.02 mmol, 0.05 equiv), and 2-[2-(2-fluoroethoxy)ethoxy]ethan-1-ol (182 mg, 1.08 mmol, 3.0 equiv) were combined in 1,4-dioxane (7.0 mL). The mixture was stirred overnight at 90 °C under N_2. After cooling to room temperature, water (30 mL) was added and the mixture was extracted with EtOAc (3 × 50 mL). The combined organic extracts were washed with brine (100 mL), dried over anhydrous

Na_2SO_4, filtered, and concentrated under reduced pressure. Purification by silica gel chromatography (DCM/MeOH, 10:1) afforded benzyl (2S)-4-{7-[(tert-butoxy)carbonyl]-2-{2-[2-(2-fluoroethoxy)ethoxy]ethoxy}-5H,6H,7H,8H-pyrido[3,4-d]pyrimidin-4-yl}-2-(cyanomethyl)piperazine-1-carboxylate as a yellow solid (150 mg, 0.20 mmol, 56.2%).

LC-MS method G: $(M+H)^+$ 643.30; Rt: 0.694 min

Step 2: benzyl (2S)-4-{7-[(tert-butoxy)carbonyl]-2-{2-[2-(2-fluoroethoxy)ethoxy] ethoxy}-5H,6H,7H,8H-pyrido[3,4-d]pyrimidin-4-yl}-2-(cyanomethyl)piperazine-1-carboxylate

Benzyl (2S)-4-{7-[(tert-butoxy)carbonyl]-2-{2-[2-(2-fluoroethoxy)ethoxy]ethoxy}-5H,6H,7H,8H-pyrido[3,4-d]pyrimidin-4-yl}-2-(cyanomethyl)piperazine-1-carboxylate (50.0 mg, 0.07 mmol, 1.0 equiv) was dissolved in DCM (1.0 mL) and treated with TFA (0.20 mL, 2.67 mmol, 39.8 equiv). The mixture was stirred at room temperature for 2 h and then concentrated in vacuo. This afforded benzyl (2S)-2-(cyanomethyl)-4-(2-{2-[2-(2-fluoroethoxy)ethoxy]ethoxy}-5H,6H,7H,8H-pyrido[3,4-d]pyrimidin-4-yl)piperazine-1-carboxylate as the TFA salt (30.0 mg, 0.04 mmol, 64% yield, 94% purity) as a yellow oil, which was used directly in the next step.

LC-MS method D: $(M+H)^+$ 543.25; Rt: 0.565 min

Step 3: benzyl (2S)-2-(cyanomethyl)-4-(2-{2-[2-(2-fluoroethoxy)ethoxy]ethoxy}-7-(8-methylnaphthalen-1-yl)-5H,6H,7H,8H-pyrido[3,4-d]pyrimidin-4-yl)piperazine-1-carboxylate

A solution of benzyl (2S)-2-(cyanomethyl)-4-(2-[2-[2-(2-fluoroethoxy)ethoxy]ethoxy]-5H,6H,7H,8H-pyrido[3,4-d]pyrimidin-4-yl)piperazine-1-carboxylate; trifluoroacetic acid (700.0 mg; 0.86 mmol; 1.0 eq.),1-bromo-8-methylnaphthalene (572.8 mg; 2.57 mmol, 3.0 eq.), Cs_2CO_3 (844.1 mg; 2.57 mmol, 3.0 eq.) and RuPhos Pd G3 (36.1 mg, 0.043 mmol, 0.05 eq.) in dioxane (20.0 mL) was stirred for overnight at 100 °C under nitrogen atmosphere. The mixture was allowed to cool down to room temperature and it was diluted with water. The resulting mixture was extracted with EtOAc three times. The combined organic layers were washed with brine, dried over anhydrous Na_2SO_4, filtered and concentrated under reduced pressure. The residue was purified by silica gel column chromatography, eluted with PE/EtOAc (1:2), to afford benzyl (2S)-2-(cyanomethyl)-4-(2-[2-[2-(2-fluoroethoxy)ethoxy]ethoxy]-7-(8-methylnaphthalen-1-yl)-5H,6H,8H-pyrido[3,4-d]pyrimidin-4-yl)piperazine-1-carboxylate (600.0 mg; 0.81 mmol; 94% yield; 92% purity) as a yellow oil.

LC-MS method D: $(M+H)^+$ 683.35; Rt: 0.670 min

Step 4: 2-[(2S)-4-(2-{2-[2-(2-fluoroethoxy)ethoxy]ethoxy}-7-(8-methylnaphthalen-1-yl)-5H,6H,7H,8H-pyrido[3,4-d]pyrimidin-4-yl)piperazin-2-yl]acetonitrile

Benzyl (2S)-2-(cyanomethyl)-4-(2-[2-[2-(2-fluoroethoxy)ethoxy]ethoxy]-7-(8-methylnaphthalen-1-yl)-5H,6H,8H-pyrido[3,4-d]pyrimidin-4-yl)piperazine-1-carboxylate (120.0 mg, 0.16 mmol, 1.0 equiv) and Pd/C (60.0 mg, 0.056 mmol, 0.4 equiv) were suspended in EtOH (1.0 mL) and THF (1.0 mL). The mixture was stirred under a hydrogen atmosphere at room temperature for 2 h. The reaction was filtered and the catalyst was rinsed with MeOH (3×). The combined filtrates were concentrated under reduced pressure to afford 2-[(2S)-4-(2-[2-[2-(2-fluoroethoxy)ethoxy]ethoxy]-7-(8-methylnaphthalen-1-yl)-5H,6H,8H-pyrido[3,4-d]pyrimidin-4-yl)piperazin-2-yl]acetonitrile as a yellow oil (80.0 mg, 0.12 mmol, 73% yield, 80% purity).

LC-MS method D: (M+H) 549.25; Rt: 0.622 min

Step 5: 2-[(2S)-4-(2-{2-[2-(2-fluoroethoxy)ethoxy]ethoxy}-7-(8-methylnaphthalen-1-yl)-5H,6H,7H,8H-pyrido[3,4-d]pyrimidin-4-yl)-1-(prop-2-enoyl)piperazin-2-yl]acetonitrile

A solution of 2-[(2S)-4-(2-[2-[2-(2-fluoroethoxy)ethoxy]ethoxy]-7-(8-methylnaphthalen-1-yl)-5H,6H,8H-pyrido[3,4-d]pyrimidin-4-yl)piperazin-2-yl]acetonitrile (75.0 mg; 0.11 mmol; 1.0 eq.), DIEA (96 µL; 0.55 mmol; 5.0 eq.) and acryloyl chloride (27 µL; 0.33 mmol; 3.0 eq) in DCM (7.0 mL) was stirred for 1h at room temperature under nitrogen atmosphere. The resulting mixture was diluted with water and extracted with EtOAc three times. The combined organic layers were washed with brine, dried over anhydrous Na_2SO_4, filtered and concentrated under reduced pressure. The crude product was purified by Prep-HPLC (XBridge Prep OBD C18 Column, 30x150mm 5 µm; mobile phase: water (10mM NH_4HCO_3 + 0.1% $NH_3.H_2O$) and ACN (35% phase B up to 68% in 8 min)) to afford 2-[(2S)-4-(2-[2-[2-(2-fluoroethoxy)ethoxy]ethoxy]-7-(8-methylnaphthalen-1-yl)-5H,6H,8H-pyrido[3,4-d]pyrimidin-4-yl)-1-(prop-2-enoyl)piperazin-2-yl]acetonitrile (14.0 mg; 0.02 mmol; 21% yield; 100% purity) as an off-white solid.

LC-MS method A: $(M+H)^+$ 603.3; Rt: 1.349 min

^1H NMR (300 MHz, DMSO-d_6): δ= 7.75 (d, J = 8.1 Hz, 1H), 7.70 (dd, J = 8.0, 3.6 Hz, 1H), 7.53 – 7.09 (m, 4H), 6.86 (br, 1H), 6.19 (dd, J = 16.7, 1.9 Hz, 1H), 5.78 (dd, J = 10.4, 2.2 Hz, 1H), 5.02 – 4.69 (m, 1H), 4.64 – 4.52 (m, 1H), 4.46 – 4.38 (m, 1H), 4.32 (q, J = 4.7 Hz, 2H), 4.12 – 3.80 (m, 4H), 3.81 – 3.61 (m, 4H), 3.60 – 3.43 (m, 5H), 3.21 – 2.96 (m, 5H), 2.86 (s, 3H). 4H are missing due to overlapping.

^{13}C NMR (101 MHz, $CDCl_3$) δ 166.17, 166.15, 166.13, 166.00, 136.35, 134.54, 130.05, 129.96, 129.92, 129.37, 129.27, 127.31, 127.28, 126.51, 125.60, 125.35, 124.85, 119.18, 84.04, 82.34, 77.22, 70.78, 70.73, 70.45, 70.27, 69.13, 50.18, 50.11, 47.98, 29.71, 26.50, 26.40, 24.14, 23.95, 18.26.

HRMS (ESI): calculated for $C_{33}H_{39}FN_6O_4$ [M+H]: 603.3090; found 603.3096

Precursor KRAS490: Example 2: 2-{2-[2-({4-[(3S)-3-(cyanomethyl)-4-(prop-2-enoyl)piperazin-1-yl]-7-(8-methylnaphthalen-1-yl)-5H,6H,7H,8H-pyrido[3,4-d]pyrimidin-2-yl}oxy)ethoxy]ethoxy}ethyl 4-methylbenzene-1-sulfonate

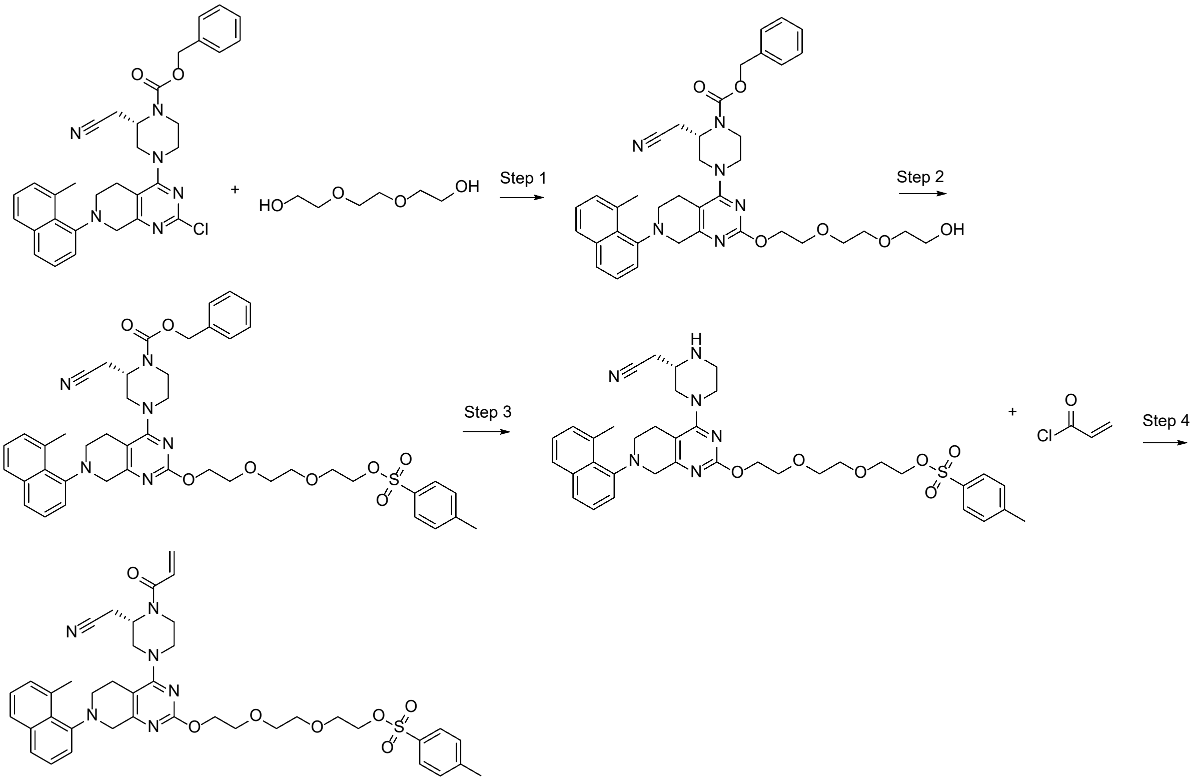

Step 1: benzyl (2S)-2-(cyanomethyl)-4-(2-{2-[2-(2-hydroxyethoxy)ethoxy]ethoxy}-7-(8-methylnaphthalen-1-yl)-5H,6H,7H,8H-pyrido[3,4-d]pyrimidin-4-yl)piperazine-1-carboxylate

Benzyl (2S)-4-[2-chloro-7-(8-methylnaphthalen-1-yl)-5H,6H,8H-pyrido[3,4-d]pyrimidin-4-yl]-2-(cyanomethyl)piperazine-1-carboxylate (280.0 mg, 0.49 mmol, 1.0 equiv), Cs_2CO_3 (482.6 mg, 1.47 mmol, 3.0 equiv), and 2-[2-(2-hydroxyethoxy)ethoxy]ethan-1-ol (741.5 mg, 4.89 mmol, 10.0 equiv) were combined in dioxane (4.0 mL) under N_2 and heated at 80 °C for 2 h. After cooling, the mixture was diluted with water and extracted with EtOAc (3×). The combined organic extracts were washed with brine, dried over anhydrous Na_2SO_4, filtered, and concentrated under reduced pressure. The crude product was purified by preparative TLC (PE/EtOAc, 1:3) to give benzyl (2S)-2-(cyanomethyl)-4-(2-[2-[2-(2-hydroxyethoxy)ethoxy]ethoxy]-7-(8-methylnaphthalen-1-yl)-5H,6H,8H-pyrido[3,4-d]pyrimidin-4-yl)piperazine-1-carboxylate as a yellow solid (340.0 mg, 0.49 mmol, 51% yield, 98% purity).

LC-MS method D: $(M+H)^+$ 681.3; Rt: 0.743 min

Step 2: benzyl (2S)-2-(cyanomethyl)-4-{2-[2-(2-{2-[(4-methylbenzenesulfonyl) oxy]ethoxy}ethoxy)ethoxy]-7-(8-methylnaphthalen-1-yl)-5H,6H,7H,8H-pyrido[3,4-d]pyrimidin-4-yl}piperazine-1-carboxylate

A solution of benzyl (2S)-2-(cyanomethyl)-4-(2-[2-[2-(2-hydroxyethoxy)ethoxy]ethoxy]-7-(8-methylnaphthalen-1-yl)-5H,6H,8H-pyrido[3,4-d]pyrimidin-4-yl)piperazine-1-carboxylate (640.0 mg; 0.92 mmol; 1.0 eq.), TEA (391 µL; 2.78 mmol; 3.0 eq.), 4-methylbenzene-1-sulfonyl chloride (268.0 mg; 1.39 mmol; 1.5 eq.) and DMAP (13.0 mg; 0.11 mmol; 0.1 eq.) in DCM (12.0 mL) was stirred for 16 h at room temperature under nitrogen atmosphere. The resulting mixture was concentrated under vacuum and the residue was purified by prep-TLC (PE/EtOAc 1:2) to afford benzyl (2S)-2-(cyanomethyl)-4-[2-[2-(2-[2-[(4-methylbenzenesulfonyl)oxy]ethoxy] ethoxy)ethoxy]-7-(8-methylnaphthalen-1-yl)-5H,6H,8H-pyrido[3,4-d]pyrimidin-4-yl]piperazine-1-carboxylate (600.0 mg; 0.68 mmol; 73% yield; 94% purity) as a yellow solid.

LC-MS method D: $(M+H)^+$ 835.3; Rt: 0.795 min

Step 3: 2-{2-[2-({4-[(3S)-3-(cyanomethyl)piperazin-1-yl]-7-(8-methylnaphthalen-1-yl)-5H,6H,7H,8H-pyrido[3,4-d]pyrimidin-2-yl}oxy)ethoxy]ethoxy}ethyl 4-methylbenzene-1-sulfonate

Benzyl (2S)-2-(cyanomethyl)-4-[2-[2-(2-[2-[(4-methylbenzenesulfonyl)oxy]ethoxy]ethoxy)ethoxy]-7-(8-methylnaphthalen-1-yl)-5H,6H,8H-pyrido[3,4-d]pyrimidin-4-yl]piperazine-1-carboxylate (600.0 mg, 0.68 mmol, 1.0 equiv) and 10% Pd/C (600.0 mg, 0.56 mmol, 0.9 equiv) were suspended in MeOH (5.0 mL) and THF (5.0 mL). The mixture was stirred under H_2 at room temperature for 2 h. The reaction was filtered to remove the catalyst, which was washed with DCM. The combined filtrates were concentrated under reduced pressure to afford 2-[2-[2-([4-[(3S)-3-(cyanomethyl)piperazin-1-yl]-7-(8-methylnaphthalen-1-yl)-5H,6H,8H-pyrido[3,4-d]pyrimidin-2-yl]oxy)ethoxy]ethoxy]ethyl 4-methylbenzenesulfonate as a yellow solid (480.0 mg, 0.64 mmol, 95% yield, 94% purity). The crude material was used directly in the subsequent step.

LC-MS method F: $(M+H)^+$ 701.4; Rt: 1.041 min

Step 4: 2-{2-[2-({4-[(3S)-3-(cyanomethyl)-4-(prop-2-enoyl)piperazin-1-yl]-7-(8-methylnaphthalen-1-yl)-5H,6H,7H,8H-pyrido[3,4-d]pyrimidin-2-yl}oxy)ethoxy]ethoxy}ethyl 4-methylbenzene-1-sulfonate

A solution of 2-[2-[2-([4-[(3S)-3-(cyanomethyl)piperazin-1-yl]-7-(8-methylnaphthalen-1-yl)-5H,6H,8H-pyrido[3,4-d]pyrimidin-2-yl]oxy)ethoxy]ethoxy]ethyl 4-methylbenzenesulfonate (430.0 mg; 0.60 mmol; 1.0 eq.), DIEA (526 µL; 2.99 mmol; 5.0 eq.) and prop-2-enoyl chloride (49 µL; 0.60 mmol; 1.0 eq.) in DCM (15.0 mL) was stirred for 30 min at room temperature under

nitrogen atmosphere. The resulting mixture was concentrated under vacuum. The residue was purified by silica gel column chromatography, eluted with PE/EtOAc (1:100), to afford 2-[2-[2-([4-[(3S)-3-(cyanomethyl)-4-(prop-2-enoyl)piperazin-1-yl]-7-(8-methylnaphthalen-1-yl)-5H,6H,8H-pyrido[3,4-d]pyrimidin-2-yl]oxy)ethoxy]ethoxy]ethyl 4-methylbenzenesulfonate (274.7 mg; 0.36 mmol; 60% yield; 99% purity) as an off-white solid.

LC-MS method A: $(M+H)^+$ 755.4; Rt: 1.554 min

^{1}H NMR (400 MHz, DMSO-d_6): δ= 7.83 – 7.71 (m, 3H), 7.73 – 7.65 (m, 1H), 7.50 – 7.41 (m, 3H), 7.40 – 7.29 (m, 2H), 7.29 – 7.20 (m, 1H), 6.85 (br, 1H), 6.19 (d, J = 16.6 Hz, 1H), 5.77 (dd, J = 10.4, 2.3 Hz, 1H), 5.06 – 4.66 (m, 1H), 4.41 – 4.21 (m, 2H), 4.10 (ddd, J = 4.5, 3.0, 1.5 Hz, 2H), 4.05 – 3.82 (m, 3H), 3.76 – 3.63 (m, 3H), 3.60 – 3.52 (m, 2H), 3.51 – 3.38 (m, 5H), 3.30 (s, 1H), 3.21 – 2.96 (m, 2H), 2.86 (s, 3H), 2.38 (s, 3H); 6H are missing due to overlapping.

^{13}C NMR (126 MHz, DMSO) δ = 161.26, 149.64, 149.36, 144.25, 135.32, 135.28, 133.48, 133.42, 131.79, 129.50, 128.99, 128.13, 128.03, 127.40, 127.00, 126.57, 125.08, 125.03, 124.88, 124.84, 118.44, 117.74, 107.90, 69.35, 69.18, 69.06, 69.04, 68.09, 68.06, 67.28, 64.97, 64.89, 59.09, 49.46, 49.37, 25.02, 22.92, 22.83, 20.45, 20.16.

HRMS (ESI): calculated for $C_{40}H_{46}N_6O_7S$ [M+H]: 755.3222; found 755.3227]

KRAS8125: Example 3: 2-[(2S)-4-[7-(8-chloronaphthalen-1-yl)-2-{2-[2-(2-fluoroethoxy)ethoxy]ethoxy}-5H,6H,7H,8H-pyrido[3,4-d]pyrimidin-4-yl]-1-(2-fluoroprop-2-enoyl)piperazin-2-yl]acetonitrile

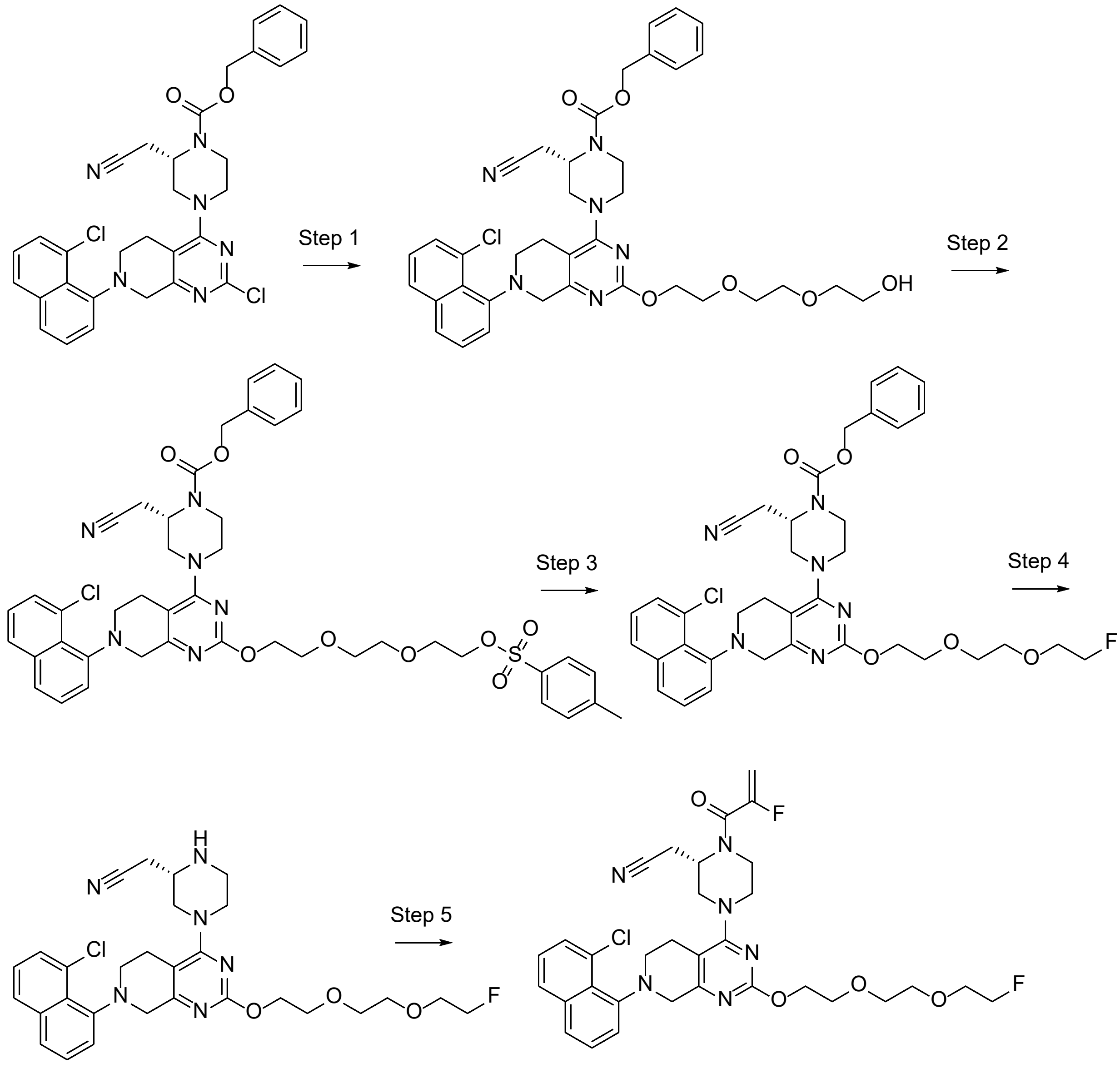

Step 1: benzyl (2S)-4-[7-(8-chloronaphthalen-1-yl)-2-{2-[2-(2-hydroxyethoxy) ethoxy]ethoxy}-5H,6H,7H,8H-pyrido[3,4-d]pyrimidin-4-yl]-2-(cyanomethyl) piperazine-1-carboxylate

Benzyl (2S)-4-[2-chloro-7-(8-chloronaphthalen-1-yl)-5H,6H,7H,8H-pyrido[3,4-d]pyrimidin-4-yl]-2-(cyanomethyl)piperazine-1-carboxylate (12.00 g, 20.43 mmol, 1.0 equiv), 2-[2-(2-hydroxyethoxy)ethoxy]ethan-1-ol (8.40 mL, 62.56 mmol, 3.1 equiv), and Cs_2CO_3 (13.20 g, 40.11 mmol, 2.0 equiv) were combined in 1,4-dioxane (120 mL) under N_2 and heated at 80 °C for 1 h. The reaction mixture was cooled to room temperature and partitioned between EtOAc and water. The organic phase was dried over anhydrous Na_2SO_4, filtered, and concentrated under reduced pressure to afford benzyl (2S)-4-[7-(8-chloronaphthalen-1-yl)-2-{2-[2-(2-hydroxyethoxy)ethoxy]ethoxy}-5H,6H,7H,8H-pyrido[3,4-d]pyrimidin-4-yl]-2-

(cyanomethyl)piperazine-1-carboxylate as a black oil (13.20 g, 13.24 mmol, 65% yield, 70% purity), which was used directly in the next step.

LC-MS method B: $(M+H)^+$ 701.2; Rt: 1.045 min

Step 2: benzyl (2S)-4-[7-(8-chloronaphthalen-1-yl)-2-[2-(2-{2-[(4-methylbenzene sulfonyl)oxy]ethoxy}ethoxy)ethoxy]-5H,6H,7H,8H-pyrido[3,4-d]pyrimidin-4-yl]-2-(cyanomethyl)piperazine-1-carboxylate

To a stirred mixture of benzyl (2S)-4-[7-(8-chloronaphthalen-1-yl)-2-{2-[2-(2-hydroxyethoxy)ethoxy]ethoxy}-5H,6H,7H,8H-pyrido[3,4-d]pyrimidin-4-yl]-2-(cyanomethyl)piperazine-1-carboxylate (12.00 g; 12.03 mmol; 1.0 eq.), Et_3N (60.0 mL) and DMAP (422 mg; 3.61 mmol; 0.3 eq.) in DCM (180 mL) TsCl (7.25 g; 36.13 mmol; 3.0 eq.) was added at 0 °C. The resulting mixture was stirred at room temperature for 3 h. The reaction mixture was concentrated under reduced pressure and the residue was purified by reverse phase chromatography (C18 silica gel column; mobile phase A: 5mM NH_4HCO_3/water and B: MeCN; gradient: 50% to 100% in 40 min) to afford benzyl (2S)-4-[7-(8-chloronaphthalen-1-yl)-2-[2-(2-{2-[(4-methylbenzenesulfonyl)oxy] ethoxy}ethoxy)ethoxy]-5H,6H,7H,8H-pyrido[3,4-d]pyrimidin-4-yl]-2-(cyanomethyl) piperazine-1-carboxylate (3.21 g; 3.73 mmol; 31% yield; 99% purity) as a yellow solid.

LC-MS method K: $(M+H)^+$ 701.2; Rt: 1.045 min

Step 3: benzyl (2S)-4-[7-(8-chloronaphthalen-1-yl)-2-{2-[2-(2-fluoroethoxy) ethoxy]ethoxy}-5H,6H,7H,8H-pyrido[3,4-d]pyrimidin-4-yl]-2-(cyanomethyl) piperazine-1-carboxylate

A mixture of benzyl (2S)-4-[7-(8-chloronaphthalen-1-yl)-2-[2-(2-{2-[(4-methylbenzene sulfonyl)oxy]ethoxy}ethoxy)ethoxy]-5H,6H,7H,8H-pyrido[3,4-d]pyrimidin-4-yl]-2-(cyanomethyl)piperazine-1-carboxylate (700.0 mg; 0.81 mmol; 1.0 eq.) in THF (17.5 mL) was stirred for 5 min at room temperature under nitrogen atmosphere. To the above mixture TBAF in THF (3.50 ml; 3.50 mmol; 4.4 eq.) was added at 0 °C. The reaction mixture was then stirred for an additional 2 h at 50 °C. The resulting mixture was cooled to room temperature and concentrated under reduced pressure. The residue was purified by reverse phase chromatography: C18 silica gel column; mobile phase A: water (0.1% FA) and B: MeCN; gradient: hold on 5% for 20 min, then 5% to 100% in 30 min) to afford benzyl (2S)-4-[7-(8-chloronaphthalen-1-yl)-2-{2-[2-(2-fluoroethoxy)ethoxy]ethoxy}-5H,6H,7H,8H-pyrido[3,4-d]pyrimidin-4-yl]-2-(cyanomethyl)piperazine-1-carboxylate (320.0 mg; 0.46 mmol; 57% yield; 100% purity) as a brown solid.

LC-MS method C: $(M+H)^+$ 703.4; Rt: 0.843 min

Step 4: 2-[(2S)-4-[7-(8-chloronaphthalen-1-yl)-2-{2-[2-(2-fluoroethoxy)ethoxy] ethoxy}-5H,6H,7H,8H-pyrido[3,4-d]pyrimidin-4-yl]piperazin-2-yl]acetonitrile

A suspension of benzyl (2S)-4-[7-(8-chloronaphthalen-1-yl)-2-{2-[2-(2-fluoroethoxy)ethoxy]ethoxy}-5H,6H,7H,8H-pyrido[3,4-d]pyrimidin-4-yl]-2-(cyanomethyl)piperazine-1-carboxylate (300.0 mg, 0.43 mmol, 1.0 equiv) and Pd/C (150.0 mg, 0.3 equiv) in MeOH (15.0 mL) and methanolic ammonia (15.0 mL) was stirred under hydrogen (H_2 balloon) at ambient temperature for 5 h. The mixture was passed through Celite to remove the catalyst and the Celite bed was washed with MeOH. Evaporation of the filtrate in vacuo provided 2-[(2S)-4-[7-(8-chloronaphthalen-1-yl)-2-{2-[2-(2-fluoroethoxy)ethoxy]ethoxy}-5H,6H,7H,8H-pyrido[3,4-d]pyrimidin-4-yl]piperazin-2-yl]acetonitrile (180.0 mg, 0.24 mmol, 55% yield, 74% purity) as a yellow solid.

LC-MS method I: $(M+H)^+$ 569.4; Rt: 0.708 min

Step 5: 2-[(2S)-4-[7-(8-chloronaphthalen-1-yl)-2-{2-[2-(2-fluoroethoxy)ethoxy] ethoxy}-5H,6H,7H,8H-pyrido[3,4-d]pyrimidin-4-yl]-1-(2-fluoroprop-2-enoyl)piperazin-2-yl]acetonitrile

A solution of 2-[(2S)-4-[7-(8-chloronaphthalen-1-yl)-2-{2-[2-(2-fluoroethoxy)ethoxy]ethoxy}-5H,6H,7H,8H-pyrido[3,4-d]pyrimidin-4-yl]piperazin-2-yl]acetonitrile (180.0 mg, 0.24 mmol, 1.0 equiv) and 2-fluoroprop-2-enoic acid (90.0 mg, 4.1 equiv) in EtOAc (9.0 mL) was prepared under nitrogen and cooled to −40 °C. Et_3N (0.25 mL, 7.9 equiv) and 50% T3P in EtOAc (0.72 mL, 5.2 equiv) were added, and the reaction mixture was stirred for 2 h while warming to room temperature. The mixture was worked up by partitioning between aqueous $NaHCO_3$ and EtOAc. The organic layer was washed with water, dried (Na_2SO_4), filtered, and evaporated in vacuo. The crude product was purified by C18 reverse-phase chromatography (A: H_2O, 0.1% NH_4CO_3; B: MeCN; 30–60% B over 30 min) to yield 2-[(2S)-4-[7-(8-chloronaphthalen-1-yl)-2-{2-[2-(2-fluoroethoxy)ethoxy]ethoxy}-5H,6H,7H,8H-pyrido[3,4-d]pyrimidin-4-yl]-1-(2-fluoroprop-2-enoyl)piperazin-2-yl]acetonitrile (43.0 mg, 0.06 mmol, 28% yield, 97% purity) as a white solid.

LC-MS method B: $(M+H)^+$ 641.2; Rt: 1.051 min

^{1}H NMR (400 MHz, DMSO-d_6): δ= 7.92 (d, J = 8.4 Hz, 1H), 7.74 (dd, J = 8.2, 3.5 Hz, 1H), 7.58 (d, J = 7.3 Hz, 1H), 7.53 (q, J = 7.5 Hz, 1H), 7.45 (t, J = 7.8 Hz, 1H), 7.34 (dd, J = 14.9, 7.5 Hz, 1H), 5.39 (dd, J = 18.0, 4.1 Hz, 1H), 5.27 (d, J = 50.9 Hz, 1H), 4.59 – 4.54 (m, 1H), 4.47 – 4.42 (m, 1H), 4.32 (q, J = 4.5 Hz, 2H), 4.18 (dd, J = 17.4, 10.6 Hz, 1H), 4.12 – 4.00 (m, 1H), 3.97 (d, J = 13.1 Hz, 1H), 3.90 (d, J = 12.7 Hz, 1H), 3.85 – 3.63 (m, 5H), 3.61 – 3.55 (m, 5H), 3.53 – 3.44 (m, 2H), 3.27 – 3.19 (m, 1H), 3.18 – 2.99 (m, 4H), 2.92 (dd, J = 17.0, 6.0 Hz, 1H), 2.68 (d, J = 13.2 Hz, 1H).

^{13}C NMR (126 MHz, DMSO) δ = 165.29, 165.27, 163.80, 163.63, 161.32, 161.29, 153.87, 147.44, 136.41, 128.90, 128.27, 127.98, 126.28, 125.34, 124.35, 124.14, 118.25, 117.53, 107.98, 83.07, 81.75, 69.23, 69.19, 69.17, 69.15, 69.00, 68.10, 64.98, 58.06, 49.43, 24.73, 24.39.

HRMS (ESI): calculated for $C_{32}H_{35}ClF_2N_6O_4$ [M+H]: 641.2450; found 641.2457

Precursor KRAS8125: Example 4: 2-[2-(2-{[7-(8-chloronaphthalen-1-yl)-4-[(3S)-3-(cyanomethyl)-4-(2-fluoroprop-2-enoyl)piperazin-1-yl]-5H,6H,7H,8H-pyrido[3,4-d]pyrimidin-2-yl]oxy}ethoxy)ethoxy]ethyl 4-methylbenzene-1-sulfonate

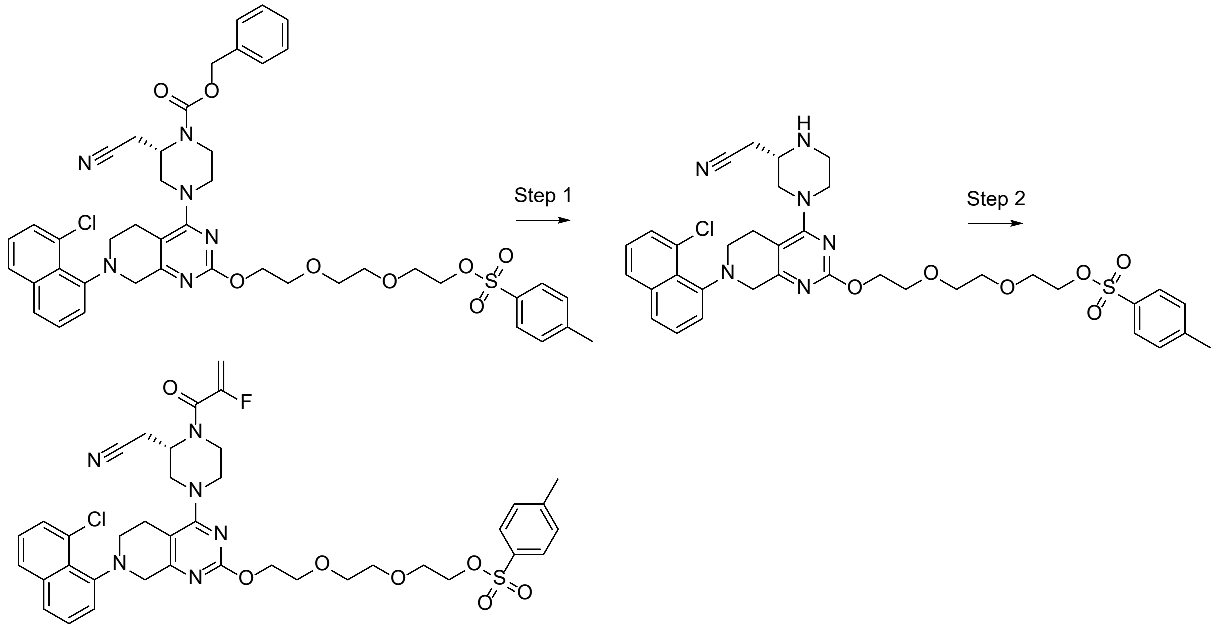

Step 1: 2-[2-(2-{[7-(8-chloronaphthalen-1-yl)-4-[(3S)-3-(cyanomethyl)piperazin-1-yl]-5H,6H,7H,8H-pyrido[3,4-d]pyrimidin-2-yl]oxy}ethoxy)ethoxy]ethyl 4-methylbenzene-1-sulfonate

A suspension of benzyl (2S)-4-[7-(8-chloronaphthalen-1-yl)-2-[2-(2-{2-[(4-methylbenzenesulfonyl)oxy]ethoxy}ethoxy)ethoxy]-5H,6H,7H,8H-pyrido[3,4-d]pyrimidin-4-yl]-2-(cyanomethyl)piperazine-1-carboxylate (500.0 mg, 0.58 mmol, 1.0 equiv) and Pd/C (250.0 mg, 0.4 equiv) in MeOH (25.0 mL) and NH_3/MeOH (25.0 mL) was stirred under hydrogen (H_2 balloon) at ambient temperature for 6 h. The reaction mixture was passed through a Celite pad to remove the catalyst and the pad was rinsed with MeOH. Removal of the solvent in vacuo furnished 2-[2-(2-{[7-(8-chloronaphthalen-1-yl)-4-[(3S)-3-(cyanomethyl)piperazin-1-yl]-5H,6H,7H,8H-pyrido[3,4-d]pyrimidin-2-yl]oxy}ethoxy)ethoxy]ethyl 4-methylbenzene-1-sulfonate (340.0 mg, 0.42 mmol, 73% yield, 90% purity) as a yellow solid, which was carried in the next step without further purification.

LC-MS method I: $(M+H)^+$ 721.4; Rt: 0.802 min

Step 2: 2-[2-(2-{[7-(8-chloronaphthalen-1-yl)-4-[(3S)-3-(cyanomethyl)-4-(2-fluoroprop-2-enoyl)piperazin-1-yl]-5H,6H,7H,8H-pyrido[3,4-d]pyrimidin-2-yl]oxy}ethoxy)ethoxy]ethyl 4-methylbenzene-1-sulfonate

A solution of 2-[2-(2-{[7-(8-chloronaphthalen-1-yl)-4-[(3S)-3-(cyanomethyl)piperazin-1-yl]-5H,6H,7H,8H-pyrido[3,4-d]pyrimidin-2-yl]oxy}ethoxy)ethoxy]ethyl 4-methylbenzene-1-sulfonate (440.0 mg, 0.61 mmol, 1.0 equiv) and 2-fluoroprop-2-enoic acid (170.0 mg, 3.0 equiv) in EtOAc (6.0 mL) was prepared under nitrogen and cooled to −40 °C. Et_3N (0.50 mL, 6.0 equiv) and 50% T3P in EtOAc (1.45 mL, 4.0 equiv) were then added, and the reaction was allowed to proceed for 2 h. The mixture was worked up by addition of saturated $NaHCO_3$ solution followed by extraction with EtOAc (3×). The combined organic phases were washed with brine, dried (Na_2SO_4), filtered, and evaporated in vacuo. The crude product was purified by C18 reverse-phase chromatography using H_2O (5 mM NH_4HCO_3) and MeCN (50–100% MeCN over 45 min) to give 2-[2-(2-{[7-(8-chloronaphthalen-1-yl)-4-[(3S)-3-(cyanomethyl)-4-(2-fluoroprop-2-enoyl)piperazin-1-yl]-5H,6H,7H,8H-pyrido[3,4-d]pyrimidin-2-yl]oxy}ethoxy)ethoxy]ethyl 4-methylbenzene-1-sulfonate (184.7 mg, 0.23 mmol, 37% yield, 98% purity) as a yellow solid.

LC-MS method K: $(M+H)^+$ 793.3; Rt: 1.377 min

^{1}H NMR (400 MHz, DMSO-d_6): δ= 7.92 (d, J = 8.1 Hz, 1H), 7.83 – 7.69 (m, 3H), 7.65 – 7.49 (m, 2H), 7.49 – 7.39 (m, 3H), 7.34 (dd, J = 14.6, 7.5 Hz, 1H), 5.39 (dd, J = 18.0, 4.1 Hz, 1H), 5.27 (d, J = 50.4 Hz, 1H), 4.85 (br, 1H), 4.30 (q, J = 4.9 Hz, 2H), 4.19 - 4.15 (m, 1H), 4.16 – 4.02 (m, 2H), 4.09 – 3.85 (m, 3H), 3.75 (t, *J* = 16.6 Hz, 1H), 3.70 – 3.63 (m, 3H), 3.57 (t, J = 4.4 Hz, 2H), 3.53 - 3.41 (m, 5H), 3.23 (dd, J = 13.8, 3.7 Hz, 1H), 3.14 - 3.05 (m, 3H), 2.91 (dd, J = 17.0, 6.0 Hz, 1H), 2.68 (d, J = 13.3 Hz, 1H), 2.38 (s, 3H). 1H is missing due to overlapping.

^{13}C NMR (126 MHz, DMSO-d_6): δ= 165.28, 163.61, 161.31, 161.28, 155.99, 153.87, 147.44, 147.24, 144.25, 136.44, 136.42, 131.79, 129.50, 128.92, 128.90, 128.27, 128.23, 128.06, 127.98, 127.40, 127.00, 126.97, 126.28, 125.35, 124.88, 124.35, 124.30, 124.16, 124.10, 118.22, 117.59, 117.52, 108.03, 69.36, 69.09, 69.05, 68.10, 68.08, 67.28, 64.97, 64.90, 64.42, 58.07, 49.42, 29.40, 24.73, 24.40, 20.46, 20.17, 18.04.

HRMS (ESI): calculated for $C_{39}H_{42}ClFN_6O_7S$[M+H]: 793.2581; found 793.2586

KRAS3776: Example 5: 2-[(2S)-4-[2-(2-fluoroethoxy)-7-(8-methylnaphthalen-1-yl)-5H,6H,7H,8H-pyrido[3,4-d]pyrimidin-4-yl]-1-(prop-2-enoyl)piperazin-2-yl]acetonitrile

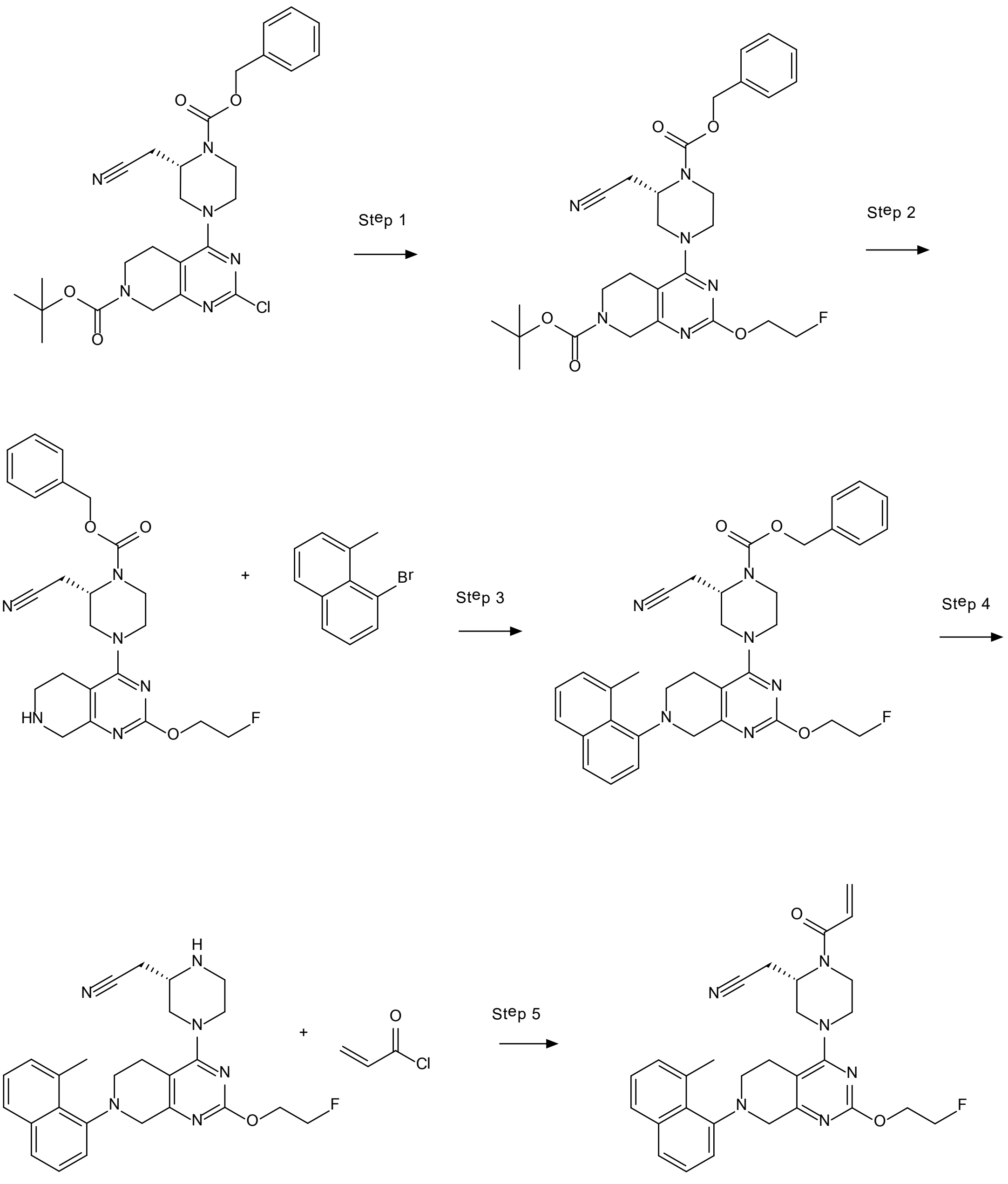

Step 1: benzyl (2S)-4-{7-[(tert-butoxy)carbonyl]-2-(2-fluoroethoxy)-5H,6H,7H,8H-pyrido[3,4-d]pyrimidin-4-yl}-2-(cyanomethyl)piperazine-1-carboxylate

To a stirred solution of benzyl (2S)-4-[7-(tert-butoxycarbonyl)-2-chloro-5H,6H,8H-pyrido[3,4-d]pyrimidin-4-yl]-2-(cyanomethyl)piperazine-1-carboxylate(500.00 mg, 0.896 mmol, 1.00 equiv, 94.4%), Cs2CO3(921.49 mg, 2.687 mmol, 3.00 equiv, 95%) and RuPhos Palladacycle Gen.3(39.42 mg, 0.045 mmol, 0.05 equiv, 95%) in dioxane (5.00 mL, 59.020 mmol, 65.90 equiv, 100%) was added 2-fluoroethanol(181.17 mg, 2.687 mmol, 3.00 equiv, 95%) . The resulting mixture was stirred for overnight at 90 degrees C under nitrogen atmosphere. The resulting mixture was diluted with H_2O (50mL) and extracted with EtOAc (3 x 50 mL). The combined organic layers were washed with brine (1x50 mL), dried over anhydrous Na2SO4. After filtration, the filtrate was concentrated under reduced pressure. The residue was purified

by Prep-TLC (PE/EtOAc 1:1) to afford benzyl (2S)-4-[7-(tert-butoxycarbonyl)-2-(2-fluoroethoxy)-5H,6H,8H-pyrido[3,4-d]pyrimidin-4-yl]-2-(cyanomethyl)piperazine-1-carboxylate(450 mg, 90.59%) as a yellow solid.

LC-MS method N: $(M+H)^+$ 555.2.1; Rt: 1.33 min

Step 2: benzyl (2S)-2-(cyanomethyl)-4-[2-(2-fluoroethoxy)-5H,6H,7H,8H-pyrido[3,4-d]pyrimidin-4-yl]piperazine-1-carboxylate hydrochloride

To a stirred solution of benzyl (2S)-4-[7-(tert-butoxycarbonyl)-2-(2-fluoroethoxy)-5H,6H,8H-pyrido[3,4-d]pyrimidin-4-yl]-2-(cyanomethyl)piperazine-1-carboxylate(450.00 mg, 0.811 mmol, 1.00 eq., 100%) in DCM (4.00 mL) was added HCl(gas) in 1,4-dioxane (1.03 mL, 28.172 mmol, 5.00 equiv, 12%). The resulting mixture was concentrated under vacuum. The resulting mixture was stirred for 1 h at room temperature. The resulting mixture was concentrated under vacuum. The crude (benzyl (2S)-2-(cyanomethyl)-4-[2-(2-fluoroethoxy)-5H,6H,7H,8H-pyrido[3,4-d]pyrimidin-4-yl]piperazine-1-carboxylate hydrochloride(420 mg,99.22 %) as a yellow solid) was carried in the next step directly without further purification.

LC-MS method E: $(M+H)^+$ 455.2.1; Rt: 0.56 min

Step 3: benzyl (2S)-2-(cyanomethyl)-4-[2-(2-fluoroethoxy)-7-(8-methylnaphthalen-1-yl)-5H,6H,7H,8H-pyrido[3,4-d]pyrimidin-4-yl]piperazine-1-carboxylate

To a stirred solution of benzyl (2S)-2-(cyanomethyl)-4-[2-(2-fluoroethoxy)-5H,6H,7H,8H-pyrido[3,4-d]pyrimidin-4-yl]piperazine-1-carboxylate hydrochloride (100.00 mg, 0.192 mmol, 1.00 equiv, 94.1%), Cs_2CO_3(197.21 mg, 0.575 mmol, 3 eq., 95%) and 1-bromo-8-methylnaphthalene (133.82 mg, 0.575 mmol, 3 eq., 95%) in dioxane (1.00 mL) was added XantPhos(49.29 mg, 0.077 mmol, 0.4 eq., 90%) and Pd_2(dba)3(39.00 mg, 0.038 mmol, 0.2 eq., 90%) . The resulting mixture was stirred for overnight at 100 degrees C under nitrogen atmosphere. The resulting mixture was diluted with H_2O (30 mL) and extracted with EtOAc (3x30 mL). The combined organic layers were washed with brine (1x30 mL), dried over anhydrous Na_2SO_4. After filtration, the filtrate was concentrated under reduced pressure. The residue was purified by Prep-TLC (PE/EtOAc 2:1) to afford benzyl (2S)-2-(cyanomethyl)-4-[2-(2-fluoroethoxy)-7-(8-methylnaphthalen-1-yl)-5H,6H,8H-pyrido[3,4-d]pyrimidin-4-yl]piperazine-1-carboxylate(90 mg, 44.38%) as a yellow solid.

LC-MS method M: $(M+H)^+$ 595.2.1; Rt: 1.16 min

Step 4: 2-[(2S)-4-[2-(2-fluoroethoxy)-7-(8-methylnaphthalen-1-yl)-5H,6H,7H,8H-pyrido[3,4-d]pyrimidin-4-yl]piperazin-2-yl]acetonitrile

To a stirred solution of benzyl (2S)-2-(cyanomethyl)-4-[2-(2-fluoroethoxy)-7-(8-methylnaphthalen-1-yl)-5H,6H,8H-pyrido[3,4-d]pyrimidin-4-yl]piperazine-1-

carboxylate(150.00 mg, 0.166 mmol, 1.00 eq., 65.9%) in THF(1.00 mL) and MeOH(1.00 mL) was added Pd/C(150.00 mg, 0.141 mmol, 0.85 eq., 10%). After completion, the mixture was passed through a filter and the solid was washed with DCM (3 × 10 mL). Removal of the solvent from the filtrate under reduced pressure gave 2-[(2R)-4-[2-(2-fluoroethoxy)-7-(8-methylnaphthalen-1-yl)-5H,6H,8H-pyrido[3,4-d]pyrimidin-4-yl]piperazin-2-yl]acetonitrile (100 mg, 93.14%) as a crude yellow solid, which was carried forward without additional purification.

LC-MS method L: $(M+H)^+$ 461.1; Rt: 0.97 min

Step 5: 2-[(2S)-4-[2-(2-fluoroethoxy)-7-(8-methylnaphthalen-1-yl)-5H,6H,7H,8H-pyrido[3,4-d]pyrimidin-4-yl]-1-(prop-2-enoyl)piperazin-2-yl]acetonitrile

A solution of 2-[(2S)-4-[2-(2-fluoroethoxy)-7-(8-methylnaphthalen-1-yl)-5H,6H,8H-pyrido[3,4-d]pyrimidin-4-yl]piperazin-2-yl]acetonitrile (90.00 mg, 0.176 mmol, 1.00 eq., 90%),DIEA (114.80 mg, 0.879 mmol, 5 eq., 99%) and acryloyl chloride (48.24 mg, 0.528 mmol, 3.00 eq., 99%) in DCM (5.00 mL) was stirred for 1h at room temperature under nitrogen atmosphere. The resulting mixture was diluted with water (30 mL). The resulting mixture was extracted with EtOAc (3x50 mL). The combined organic layers were washed with brine (1x100 mL), dried over anhydrous Na_2SO_4. After filtration, the filtrate was concentrated under reduced pressure. The crude product was purified by prep-HPLC with the following conditions: Column, XBridge Prep OBD C18 Column, 30µm,150mm 5 µm; mobile phase, Water(10mM NH_4HCO_3) and ACN (42% Phase B up to 70% in 8 min) to afford 2-[(2S)-4-[2-(2-fluoroethoxy)-7-(8-methylnaphthalen-1-yl)-5H,6H,8H-pyrido[3,4-d]pyrimidin-4-yl]-1-(prop-2-enoyl)piperazin-2-yl]acetonitrile (7.6 mg, 8.24%) as an off-white solid.

LC-MS method D: $[M+H]^+$ 515.1; Rt: 1.52 min

^{1}H NMR (300 MHz, DMSO-d6) δ= 7.81 - 7.66 (m, 2H), 7.46 - 7.28 (m, 4H), 6.87 (s, 1H), 6.20 (d, J = 16.6 Hz, 1H), 5.79 (d, J = 11.4 Hz, 1H), 5.10 - 4.63 (m, 3H), 4.51 - 4.31 (m, 3H), 4.09 - 3.92 (m, 4H), 3.73 (t, J = 17.8 Hz, 1H), 3.44 (s, 1H), 3.21 - 3.50 (m, 5H), 2.87 (s, 4H), 1.24 (s, 1H).

^{13}C NMR (126 MHz, $CDCl_3$) δ 166.34, 166.13, 165.97, 136.32, 134.59, 134.57, 129.95, 129.90, 129.37, 129.27, 127.30, 126.51, 125.60, 125.36, 125.29, 119.08, 109.16, 82.18, 80.82, 66.48, 66.32, 50.15, 50.12, 48.01, 47.80, 33.12, 31.92, 26.44, 24.14, 23.96.

HRMS (ESI): calculated for $C_{29}H_{31}FN_6O_2$ [M+H]: 515.2565; found 515.2567

Precursor KRAS3776: Example 6: 2-[(2S)-4-[2-hydroxy-7-(8-methylnaphthalen-1-yl)-5H,6H,7H,8H-pyrido[3,4-d]pyrimidin-4-yl]piperazin-2-yl]acetonitrile

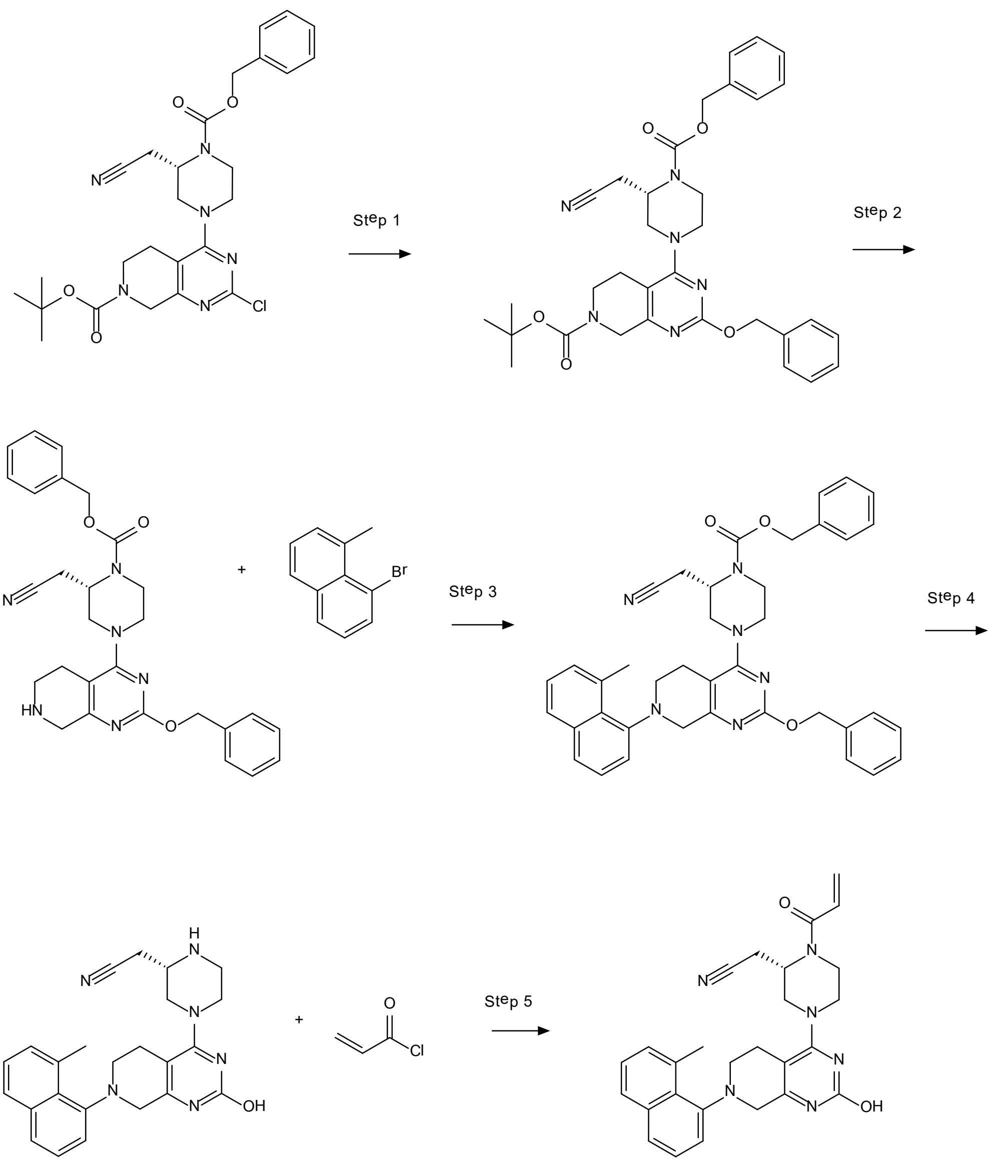

Step 1: benzyl (2S)-4-[2-(benzyloxy)-7-[(tert-butoxy)carbonyl]-5H,6H,7H,8H-pyrido[3,4-d]pyrimidin-4-yl]-2-(cyanomethyl)piperazine-1-carboxylate

A solution of benzyl (2S)-4-[7-(tert-butoxycarbonyl)-2-chloro-5H,6H,8H-pyrido[3,4-d]pyrimidin-4-yl]-2-(cyanomethyl)piperazine-1-carboxylate (6.00 g, 11.282 mmol, 1.00 eq., 99.1%),Cs_2CO_3 (11.14 g, 33.847 mmol, 3 eq., 99%),benzyl alcohol (4.93 g, 45.129 mmol, 4 eq., 99%) and RuPhos Palladacycle Gen.3 (0.48 g, 0.564 mmol, 0.05 eq., 99%) in dioxane (120mL) was stirred for 16 h at 90 °C under a nitrogen atmosphere. The residue was dissolved in EtOAc (250 mL). The resulting mixture was washed with of water (3x100 mL) and brine (300 mL). The aqueous layer was extracted with EtOAc (2x100 mL). The resulting mixture was concentrated under vacuum. The residue was purified by silica gel column chromatography, eluted with PE/EtOAc (1:1) to afford benzyl (2S)-4-[2-(benzyloxy)-7-(tert-butoxycarbonyl)-5H,6H,8H-pyrido[3,4-d]pyrimidin-4-yl]-2-(cyanomethyl)piperazine-1-carboxylate (4.4 g, 61.7%) as a yellow solid. solid

LC-MS method D: $[M+H]^+$ 599.3; Rt: 0.77 min

Step 2: benzyl (2S)-4-[2-(benzyloxy)-5H,6H,7H,8H-pyrido[3,4-d]pyrimidin-4-yl]-2-(cyanomethyl)piperazine-1-carboxylate

To a stirred solution of benzyl (2S)-4-[2-(benzyloxy)-7-(tert-butoxycarbonyl)-5H,6H,8H-pyrido[3,4-d]pyrimidin-4-yl]-2-(cyanomethyl)piperazine-1-carboxylate (4.40 g, 6.960 mmol, 1.00 eq., 94.7%) in DCM (50.00 mL) was added HCl(gas) in 1,4-dioxane (8.81 mL, 34.794 mmol, 5.00 eq., 12%) dropwiseat room temperature. The resulting mixture was stirred for 4 h at room temperature. Desired product could be detected by LCMS. After removal of volatiles under reduced pressure, the residue was dissolved in DCM (10 mL). The aqueous phase was brought to pH 8 using saturated $NaHCO_3$ solution and the product was extracted with DCM (3 × 30 mL). The pooled organic phases were washed with brine (1 × 50 mL), dried (Na_2SO_4), filtered, and evaporated in vacuo. The resulting crude product was carried forward without additional purification.

LC-MS method D: $[M+H]^+$ 499.2; Rt: 0.65 min

Step 3: benzyl (2S)-4-[2-(benzyloxy)-7-(8-methylnaphthalen-1-yl)-5H,6H,7H,8H-pyrido[3,4-d]pyrimidin-4-yl]-2-(cyanomethyl)piperazine-1-carboxylate

A solution of benzyl (2S)-4-[2-(benzyloxy)-5H,6H,7H,8H-pyrido[3,4-d]pyrimidin-4-yl]-2-(cyanomethyl)piperazine-1-carboxylate (3.00 g, 4.687 mmol, 1.00 eq., 77.9%),Cs_2CO_3 (4.63 g, 14.062 mmol, 3 eq., 99%), 1-bromo-8-methylnaphthalene (3.27 g, 14.062 mmol, 3.00 equiv, 95%),XantPhos (0.55 g, 0.937 mmol, 0.2 eq., 99%) and $Pd_2(dba)_3$ (0.43 g, 0.469 mmol, 0.1 eq., 99%) in dioxane (60.00 mL) was stirred for 16 h at 100 °C under nitrogen atmosphere. The residue was dissolved in water (150 mL). The resulting mixture was extracted with EtOAc (3x100mL). The combined organic layers were washed with brine (1x200 mL), dried over anhydrous Na_2SO_4. After filtration, the filtrate was concentrated under reduced pressure. The residue was purified by silica gel column chromatography, eluted with PE/EtOAc (2:1) to afford benzyl (2S)-4-[2-(benzyloxy)-7-(8-methylnaphthalen-1-yl)-5H,6H,8H-pyrido[3,4-d]pyrimidin-4-yl]-2-(cyanomethyl)piperazine-1-carboxylate (2.3 g, 62.91%) as a yellow oil.

LC-MS method D: $[M+H]^+$ 639.2; Rt: 0.82 min

Step 4: 2-[(2S)-4-[2-hydroxy-7-(8-methylnaphthalen-1-yl)-5H,6H,7H,8H-pyrido [3,4-d]pyrimidin-4-yl]piperazin-2-yl]acetonitrile

A suspension of benzyl (2S)-4-[2-(benzyloxy)-7-(8-methylnaphthalen-1-yl)-5H,6H,8H-pyrido[3,4-d]pyrimidin-4-yl]-2-(cyanomethyl)piperazine-1-carboxylate (2.30 g, 2.949 mmol, 1.0 equiv, 81.9%) and 10% Pd/C (2.30 g, 0.73 equiv) in MeOH (15.0 mL) was prepared at room temperature. Methanolic NH_3 (13%, 15.0 mL, 23.30 equiv) was added dropwise and the

mixture was hydrogenated at ambient temperature for 2 h. After removal of solvent under reduced pressure, the residue was taken up in DMF (3 mL) and purified by C18 reverse-phase flash chromatography using a MeCN/H_2O gradient (40–60% MeCN over 10 min). This provided 2-[(2S)-4-[2-hydroxy-7-(8-methylnaphthalen-1-yl)-5H,6H,8H-pyrido[3,4-d]pyrimidin-4-yl]piperazin-2-yl]acetonitrile (900 mg, 73.55%) as an off-white solid.

LC-MS method D: $[M+H]^+$ 415.2; Rt: 0.61 min

Step 5: 2-[(2S)-4-[2-hydroxy-7-(8-methylnaphthalen-1-yl)-5H,6H,7H,8H-pyrido [3,4-d]pyrimidin-4-yl]piperazin-2-yl]acetonitrile

A solution of 2-[(2S)-4-[2-hydroxy-7-(8-methylnaphthalen-1-yl)-5H,6H,8H-pyrido[3,4-d]pyrimidin-4-yl]piperazin-2-yl]acetonitrile (900.00 mg, 2.169 mmol, 1.00 eq., 99.9%),DIEA (1415.83 mg, 10.845 mmol, 5 eq., 99%) and prop-2-enoyl chloride (178.47 mg, 1.952 mmol, 0.9 eq., 99%) in DCM (150. mL) was stirred for 1 h at room temperature under nitrogen atmosphere. The resulting mixture was diluted with water (10mL) and extracted with DCM (3x20mL). The combined organic layers were washed with brine (1x50 mL), dried over anhydrous Na_2SO_4. After filtration, the filtrate was concentrated under reduced pressure. The resulting mixture was diluted with DMF (3mL). The residue was purified by reverse flash chromatography with the following conditions: column, C18 silica gel; mobile phase, ACN in water, 30% to 50% gradient in 10 min to afford 2-[(2S)-4-[2-hydroxy-7-(8-methylnaphthalen-1-yl)-5H,6H,8H-pyrido[3,4-d]pyrimidin-4-yl]-1-(prop-2-enoyl)piperazin-2-yl]acetonitrile
(533.7 mg, 52.4%) as an off-white solid.

LC-MS method A: $[M+H]^+$ 469.1; Rt: 1.243 min

^{1}H NMR (300 MHz, DMSO-d_6) δ= 10.91 (s, 1H), 7.82 - 7.68 (m, 2H), 7.40 (ddt, J = 30.0, 22.4, 7.1 Hz, 4H), 6.86 (s, 1H), 6.27 – 6.15 (m, 1H), 5.79 (d, J = 10.6 Hz, 1H), 4.92 (s, 1H), 4.08 – 3.91 (m, 2H), 3.91 – 3.76 (m, 2H), 3.58 (dd, J = 25.8, 17.7 Hz, 2H), 3.13 (s, 4H), 2.88 (m, 5H), 2.63 (s, 1H), 2.32 (m, 1H).

^{13}C NMR (126 MHz, DMSO-d_6) δ = 167.23, 164.36, 154.60, 149.27, 148.84, 135.23, 133.38, 129.16, 128.08, 127.32, 126.62, 125.46, 125.30, 125.10, 124.94, 118.86, 118.75, 117.70, 54.02, 53.31, 49.33, 48.60, 46.67, 45.61, 23.93, 22.93, 22.83.

HRMS (ESI): calculated for $C_{27}H_{28}N_6O_2$ M+H]: 469.2347; found 469.2347

**Example No.**	**Structure**	**Name**	**MSC**

**1**	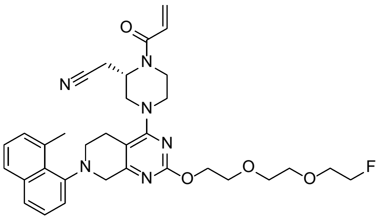	2-[(2S)-4-(2-{2-[2-(2-fluoroethoxy)ethoxy]ethoxy}-7-(8-methylnaphthalen-1-yl)-5H,6H,7H,8H-pyrido[3,4-d]pyrimidin-4-yl)-1-(prop-2-enoyl)piperazin-2-yl]acetonitrile	**KRAS490**
**2**	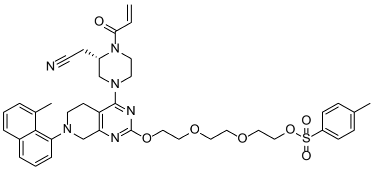	2-{2-[2-({4-[(3S)-3-(cyanomethyl)-4-(prop-2-enoyl)piperazin-1-yl]-7-(8-methylnaphthalen-1-yl)-5H,6H,7H,8H-pyrido[3,4-d]pyrimidin-2-yl}oxy)ethoxy]ethoxy}ethyl 4-methylbenzene-1-sulfonate	**Precursor KRAS490**
**3**	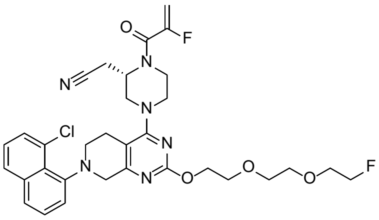	2-[(2S)-4-[7-(8-chloronaphthalen-1-yl)-2-{2-[2-(2-fluoroethoxy)ethoxy]ethoxy}-5H,6H,7H,8H-pyrido[3,4-d]pyrimidin-4-yl]-1-(2-fluoroprop-2-enoyl)piperazin-2-yl]acetonitrile	**KRAS8125**
**4**	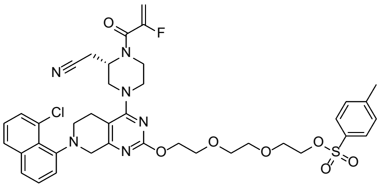	2-[2-(2-{[7-(8-chloronaphthalen-1-yl)-4-[(3S)-3-(cyanomethyl)-4-(2-fluoroprop-2-enoyl)piperazin-1-yl]-5H,6H,7H,8H-pyrido[3,4-d]pyrimidin-2-yl]oxy}ethoxy)ethoxy]ethyl 4-methylbenzene-1-sulfonate	**Precursor KRAS8125**
**5**	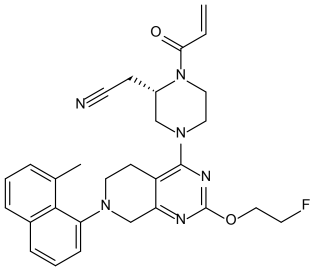	2-[(2S)-4-[2-(2-fluoroethoxy)-7-(8-methylnaphthalen-1-yl)-5H,6H,7H,8H-pyrido[3,4-d]pyrimidin-4-yl]-1-(prop-2-enoyl)piperazin-2-yl]acetonitrile	**KRAS3776**
**6**	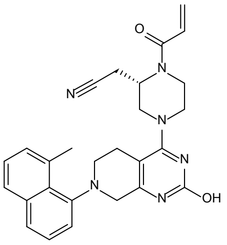	2-[(2S)-4-[2-hydroxy-7-(8-methylnaphthalen-1-yl)-5H,6H,7H,8H-pyrido[3,4-d]pyrimidin-4-yl]piperazin-2-yl]acetonitrile	**Precursor KRAS3776**

References

[1] Blake JF, Burgess LE, Chicarelli MJ, Christensen JG, Cook A, Fell JB, et al. KRAS G12C inhibitors. US Patent Application US20190144444A1. Published May 16, 2019.
